# Mixtures of Mycotoxins, Phytoestrogens, and Other Secondary Metabolites in Whole-Plant Corn Silages and Total Mixed Rations of Dairy Farms in Central and Northern Mexico

**DOI:** 10.3390/toxins15020153

**Published:** 2023-02-13

**Authors:** Felipe Penagos-Tabares, Michael Sulyok, Juan-Ignacio Artavia, Samanta-Irais Flores-Quiroz, César Garzón-Pérez, Ezequías Castillo-Lopez, Luis Zavala, Juan-David Orozco, Johannes Faas, Rudolf Krska, Qendrim Zebeli

**Affiliations:** 1 Unit of Nutritional Physiology, Institute of Physiology, Pathophysiology, and Biophysics, Department of Biomedical Sciences, University of Veterinary Medicine Vienna, Veterinärplatz 1, 1210 Vienna, Austria; 2 Christian-Doppler-Laboratory for Innovative Gut Health Concepts in Livestock (CDL-LiveGUT), Department for Farm Animals and Veterinary Public Health, University of Veterinary Medicine, Veterinaerplatz 1, 1210 Vienna, Austria; 3 FFoQSI GmbH—Austrian Competence Centre for Feed and Food Quality, Safety and Innovation, Technopark 1C, 3430 Tulln, Austria; 4 Department of Agrobiotechnology, IFA-Tulln, Institute of Bioanalytics and Agro-Metabolomics, University of Natural Resources and Life Sciences, Vienna, Konrad-Lorenz-Strasse 20, 3430 Tulln, Austria; 5 DSM-BIOMIN Research Center, Technopark 1, 3430 Tulln, Austria; 6 Facultad de Estudios Superiores Cuautitlán, Cuautitlán, Medicina Veterinaria y Zootecnia, Universidad Nacional Autónoma de México (UNAM), Cuautitlán Izcalli 54714, Mexico; 7 Institute of Animal Nutrition and Functional Plant Compounds, Department of Farm Animals and Veterinary Public Health, University of Veterinary Medicine Vienna, Veterinaerplatz 1, 1210 Vienna, Austria; 8 Institute for Global Food Security, School of Biological Sciences, Queen’s University Belfast, 19 Chlorine Gardens, Belfast BT9 5DL, UK

**Keywords:** feed safety, multi-mycotoxin analysis, dairy farming, total mixed rations, maize silage, phytoestrogens, co-occurrence

## Abstract

Mycotoxins and endocrine disruptors such as phytoestrogens can affect cattle health, reproduction, and productivity. Most studies of mycotoxins in dairy feeds in Mexico and worldwide have been focused on a few (regulated) mycotoxins. In contrast, less known fungal toxins, phytoestrogens, and other metabolites have been neglected and underestimated. This study analyzed a broad spectrum (>800) of mycotoxins, phytoestrogens, and fungal, plant, and unspecific secondary metabolites in whole-plant corn silages (WPCSs) and total mixed rations (TMRs) collected from 19 Mexican dairy farms. A validated multi-metabolite liquid chromatography/electrospray ionization–tandem mass spectrometric (LC/ESI–MS/MS) method was used. Our results revealed 125 of >800 tested (potentially toxic) secondary metabolites. WPCSs/TMRs in Mexico presented ubiquitous contamination with mycotoxins, phytoestrogens, and other metabolites. The average number of mycotoxins per TMR was 24, ranging from 9 to 31. *Fusarium*-derived secondary metabolites showed the highest frequencies, concentrations, and diversity among the detected fungal compounds. The most frequently detected mycotoxins in TMRs were zearalenone (ZEN) (100%), fumonisin B1 (FB1) (84%), and deoxynivalenol (84%). Aflatoxin B1 (AFB1) and ochratoxin A (OTA), previously reported in Mexico, were not detected. All TMR samples tested positive for phytoestrogens. Among the investigated dietary ingredients, corn stover, sorghum silage, and concentrate proportions were the most correlated with levels of total mycotoxins, fumonisins (Fs), and ergot alkaloids, respectively.

## 1. Introduction

According to the Food and Agriculture Organization (FAO), Mexico is included in the list of the countries with the highest milk deficits, along with China, Italy, the Russian Federation, Algeria, and Indonesia [1,2]. The health, productivity, and reproductive performance of dairy cattle, as well as the quality and safety of the milk, depend widely on feed quality and management [3]. In recent years, growing evidence of ubiquitous multi-mycotoxin contamination of agricultural commodities has increased interest and concern regarding the occurrence of this contaminant in multiple agriculture sectors, including dairy cattle feedstuffs and diets [2,4,5]. The dairy cow diet varies extensively among farms, seasons, and production systems worldwide, including various ingredients, primary forages, cereal grains, and agro-industrial byproducts [3]. Such diversity of feedstuffs contributes to dietary exposure to a broad spectrum of toxic and potentially toxic compounds. Among such compounds, mycotoxins have been classified as one of the riskiest substances that jeopardize feed and food safety [6,7]. Although over 500 compounds have been considered mycotoxins, most studies have investigated a limited number of mycotoxins in agricultural commodities [4,8,9,10]. Previous surveillance studies on contamination of dairy cattle feed in Mexico (via enzyme-linked immunosorbent assay and high-performance liquid chromatography) [11,12,13,14,15,16], but also worldwide, have been focused mainly on mycotoxins addressed by regulatory limits or guidance levels in animal feed—for instance, aflatoxins (AFs), zearalenone (ZEN), ochratoxin A (OTA), and trichothecenes (type A and B) [16,17,18,19].

Total mixed rations (TMR) is a popular “complete ration” feeding system used in dairy farms with large herd sizes. It is produced by mixing forages, byproducts, cereal grains, concentrates, minerals, vitamins, and other additives, supplying the nutrients needed to meet maintenance and production requirements [20,21]. TMRs have been shown to be contaminated with complex mycotoxin cocktails and other secondary metabolites from fungi, bacteria, and plants [22,23]. Additionally, whole-plant corn silage (WPCS) is one the most frequent ingredients incorporated in many countries in modern dairy and beef farming. Specifically, WPCS is the most widely used silage in North America [24,25]. It has been described in multiple regions that WPCS is one of the feedstuffs with significant relevance to the dietary contamination of mycotoxins [2,23,26,27].

Mycotoxicoses in cattle are usually ambiguous subclinical disorders affecting herds. These can occur from chronic syndromes of impaired rumen function or increased predisposition to infectious diseases and, less frequently, acute toxicoses with severe illness and death [8,28,29]. In addition, recent studies have shown that mycotoxins are highly relevant risk factors for pregnant animals. Prenatal exposure to these compounds can compromise the postnatal development of several organic systems, such as the reproductive, nervous, and circulatory systems [30,31,32,33]. Moreover, complex toxicological interactions, including addition, synergism, potentiation, and antagonism, among mycotoxins undoubtedly affect animal and human health and reproduction [34]. Such interactions between co-occurring mycotoxins demands more research and risk assessment using integrative methodologies, including the multi-mycotoxin analysis approach [2,4]. Additionally, other naturally occurring substances (for example, plant-derived metabolites such as phytoestrogens) present mainly in Leguminosae plants and can act as endocrine disruptors, impairing the reproductive performance of livestock [35,36,37,38].

Studies on the broad spectrum of mycotoxins and other endocrine disruptors of natural origin in the feedstuffs and diets of dairy cows and other food-producing animals are essential. However, they are still very limited [2,4]. Therefore, this study aimed to determine co-occurrences and concentrations of mycotoxins and other fungal metabolites (derived from the genus *Alternaria*, *Aspergillus*, *Fusarium*, *Penicillium,* other fungi, and ergot alkaloids) as well as secondary plant metabolites (such as phytoestrogens and others) in TMRs and WPCSs from large dairy cattle farms located in northern and central Mexico. The analysis was achieved using a validated multi-metabolite analysis. The possible associations of the main dietary ingredients to the levels of mycotoxins and other secondary metabolites contained in the TMRs were also assessed.

## 2. Results

### 2.1. Main Dietary Ingredients

The frequency of the inclusion and dietary levels of the main ingredients of TMRs formulated for lactating cows for all surveyed farms are presented in Table 1. The dairy farms participating in the study fed TMRs containing highly balanced proportions of forage and concentrates, averaging 49.9% and 50.1%, respectively. The forage-to-concentrate ratio (F:C) fluctuated between 40:60 and 60:40. The most common dietary components included were WPCSs (100%), alfalfa hay (79%), and high-energy density concentrate (74%). Less commonly included were protein-rich concentrate (26%), corn stover (21%), and rolled corn (21%). Other TMR ingredients with frequencies of inclusion under 20% were corn meal (16%), oat hay (16%), and sorghum silage (11%) as well as corn bran, alfalfa silage, bakery byproduct, and brewery spent grain (5%) (Table 1).

Regarding the proportion of inclusion (dietary content), commercial high-energy density concentrate was the dietary ingredient most abundant in the evaluated TMRs, with an average inclusion of 45.5% on a dry matter (DM) basis, ranging from 28.3% to 60%. WPCSs averaged 38.9% DM of the rations, varying from 27.5% to 53%. The diets that included rolled corn, corn meal, and protein-rich concentrate presented an average of 25.5%, 24.2%, and 22.6% DM, respectively. The remaining ingredients were included in the TMR formulations with an average of inclusion (proportion) less than 15% DM (Table 1).

### 2.2. Occurrence and Concentrations of the Detected Metabolites

#### 2.2.1. General Overview

**Figure 1:**
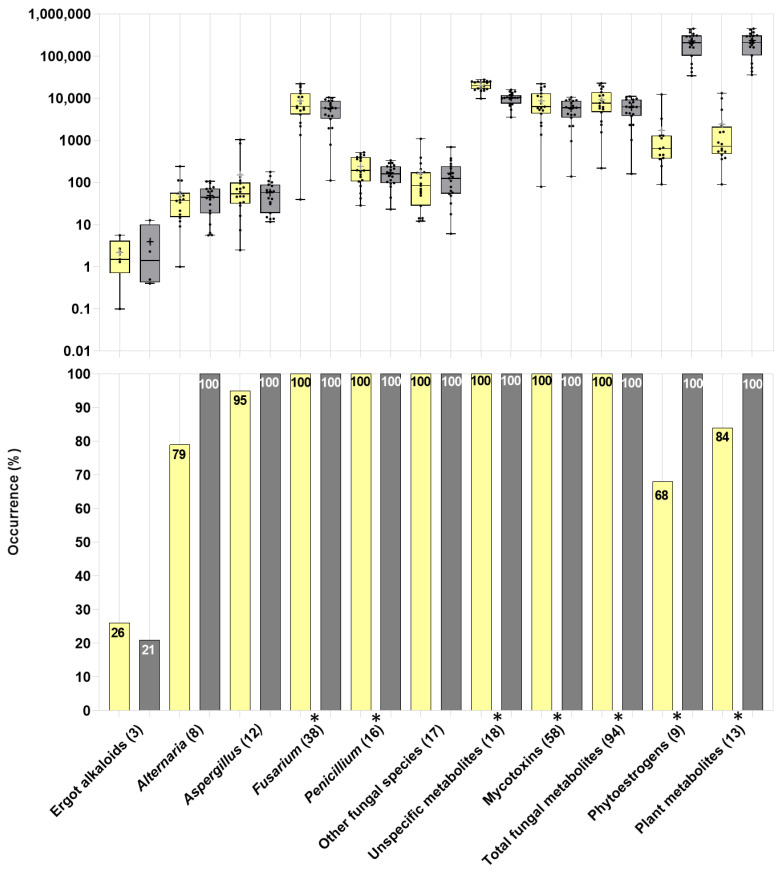
Occurrence and distribution of concentrations (µg/kg on DM basis, log 10 scale) of groups of secondary metabolites detected in whole-plant corn silages (in yellow) and total mixed rations (in gray) on dairy farms in Mexico. The total number of secondary metabolites detected per group is shown in parentheses. Asterisks (*) show significant differences (*p*-value < 0.05) between the concentrations of the respective groups in whole-plant corn silages and total mixed rations according to the Wilcoxon matched-pairs signed rank test (*p*-values in Table 2). Means are shown as “+”.

The 125 identified biological compounds in representative samples of WPCSs (114) and TMRs (118) were grouped based on their reported primary producers. These compounds consisted of 94 fungal metabolites, derived from the genera *Alternaria* (number of detected metabolites: 8), *Aspergillus* (12), *Fusarium* (38), *Penicillium* (16), or other fungi (17) and ergot-derived alkaloids (3). Thirteen compounds were plant-derived metabolites (including 9 phytoestrogens), 18 were unspecific metabolites (multi-kingdom-derived, i.e., derived from fungi, bacteria, and/or plants), and 1 was of bacterial origin (Figure 1). Figure 1 and Table 2 illustrate the occurrence and concentrations of the groups of metabolites. Additionally, Table 2 shows the significance level of a paired comparison between the WPCSs and TMRs per respective farm.

Regarding the fungal metabolites, the group of ergot alkaloids presented a minor occurrence as a group, detected in 26% of the WPCSs and 21% of the TMRs. All the TMR samples showed metabolites derived from *Alternaria*, *Aspergillus*, *Fusarium*, *Penicillium,* other fungi, phytoestrogens, and unspecific metabolites. The analyzed WPCS samples contained the ubiquitous presence of all the mentioned categories except the plant metabolites (occurrence: 84%), including phytoestrogens (68%) (Figure 1). The plant-derived metabolites (specifically, the accumulated phytoestrogens) were the compounds detected with the highest concentration in the TMRs (Figure 1, Table 2 and Table 3, Supplementary Figure S1a,b). The phytoestrogen content in the TMRs was above 34,300 µg/kg. The levels of phytoestrogens in the WPCSs were low compared to the TMRs, with a maximum content of 12,350 µg/kg. After the total plant-derived metabolites, total unspecific metabolites showed the second-highest concentrations among the groups, ranging from 9860 µg/kg to 27,790 µg/kg in the analyzed silages and from 3540 µg/kg to 15,980 µg/kg in the TMRs. Regarding the accumulated concentrations of fungal-produced metabolites, the fusarial metabolites showed the highest concentrations, with an average of 8702 µg/kg (range: 39.6 µg/kg–22,110 µg/kg) in WPCSs and 5550 µg/kg (range: 112 µg/kg–10,500 µg/kg) in TMR samples. The second most produced group of fungal metabolites was the *Penicillium*-derived compounds, which presented, on average, 243 µg/kg (range: 29 µg/kg–520 µg/kg) in the WPCSs and 171 µg/kg (range: 23.2 µg/kg–337 µg/kg) in the TMRs. Subsequently, the group of metabolites produced by other fungi presented, on average, 164 µg/kg (range: 12 µg/kg–1090 µg/kg) in WPCSs and 169 µg/kg (range: 6.1 µg/kg–697 µg/kg) in TMRs. *Aspergillus*-produced compounds were found in average levels of 154 µg/kg (range: 2.50 µg/kg–1040 µg/kg) in WPCSs and 61.7 µg/kg (range: 11.7 µg/kg–179 µg/kg) in TMRs. Compounds derived from *Alternaria* present in WPCSs fluctuated from 9.1 µg/kg to 242 µg/kg and in TMRs varied from 5.6 µg/kg to 108 µg/kg. The ergot alkaloids were detected in very low concentrations—for instance, on average, 2.24 µg/kg (maximum 5.60 µg/kg) in the silages and 4.00 µg/kg (max: 12.7 µg/kg) in the rations. The average content of total fungal metabolites in WPCSs was 9300 µg/kg (max: 22,800 µg/kg), and in TMRs it was 6001 µg/kg (max: 11,070 µg/kg). The levels of mycotoxin contamination presented a mean of 8710 µg/kg (range: 79.5 µg/kg–21,960 µg/kg) in the silages and 5590 µg/kg (range: 139 µg/kg–10,570 µg/kg) in the dietary rations (Figure 1, Supplementary Figure S1a). The concentrations of metabolites derived from *Fusarium* (*p*-value = 0.0005), *Penicillium* (*p*-value = 0.0108), total fungal metabolites (*p*-value = 0.0004), total unspecific metabolites (*p*-value < 0.0001), and accumulated mycotoxins (*p*-value = 0.0006) were significantly higher in WPCSs than in TMRs. On the contrary, the plant-derived metabolites (represented primarily by phytoestrogens) presented significantly higher (*p*-value < 0.0001) levels in the TMRs than in the silages (Figure 1, Supplementary Figure S1b, Table 2).

#### 2.2.2. Mycotoxins and Other Fungal Secondary Metabolites

Of the 94 fungal-derived metabolites detected, 58 have been previously reported as mycotoxins (Table 3). Among the major mycotoxins, ZEN, deoxynivalenol (DON), FA1, FA2, FB1, FB2, FB3, and FB4 were detected in TMRs as well as in WPCSs. The carcinogenic mycotoxins AFB1 and OTA were not detected in the assessed feed samples. Toxin metabolites related to the parent major mycotoxins such as nivalenol (NIV), DON-3-glucoside, and hydrolyzed FB1 were found in silages and in rations. 15-acetyl-deoxynivalenol was seen only in WPCSs (11%). ZEN was detected in all the TMR samples and in 68% of the WPCSs. The dietary levels of ZEN had an average of 38.7 µg/kg and a maximum concentration of 246 µg/kg. DON was also found with a higher frequency in TMRs (84%) than in WPCSs (53%). The average concentration of DON in TMRs was 615 µg/kg, and the maximum level was 1660 µg/kg. FB1 and FB2 were the most detected and with the highest levels among the fumonisins (Fs) in TMRs, with occurrences of 84% and 64% and an average of 218 µg/kg and 103 µg/kg, respectively. NIV was detected more frequently in TMRs (68%) than in WPCSs (42%). The levels of NIV were significantly higher (*p*-value = 0.0061) in samples of TMRs (mean: 872 µg/kg; max: 2600 µg/kg) than in WPCSs (mean: 269 µg/kg; max: 614 µg/kg) (Table 3). The totals of Fs (sum of FA1, FA2, FB1, FB2, FB3, FB4 hydrolyzed B1) and a total of type B trichothecenes (sum of DON, 15-acetyl-DON, DON-3-glucoside, and NIV) were superior in WPCSs than in TMRs; however, the differences were not significant. The maximum amount of total Fs in silages was 4410 µg/kg, and it was 1670 µg/kg in mixed rations. The maximum concentration of type B trichothecenes in WPCSs was 4230 µg/kg, whereas the highest concentration in the analyzed TMR samples was 5510 µg/kg. Concerning other less studied mycotoxins derived from *Fusarium* spp. such as beauvericin, beauvericin A, bikaverin, and enniatins (ENNs) (A, A1, A2, B, B1, and B2), epiquisetin, equisetin, fusaric acid, culmorin, moniliformin, and siccanol, among others, were also detected. Beauvericin, bikaverin, and moniliformin were detected in all the analyzed TMRs. Siccanol was the *Fusarium*-derived metabolite with the highest concentration, averaging 2510 µg/kg and with a maximum level of 6130 µg/kg. The second most produced fusarial metabolite was 15-hydroxyculmorin (average: 1270 µg/kg; max: 1510 µg/kg). The total content of ENNs was significantly superior (*p*-value = 0.0144) in TMRs than in WPCSs, with a respective average of 11.2 µg/kg and 5.96 µg/kg. Among mycotoxins produced by *Penicillium* spp., mycophenolic acid was detected in TMRs, with an occurrence of 42%. Citrinin, primarily *Penicillium*-derived but also produced by some Aspergilli, was seen in only 1 TMR sample (5%). Concerning the metabolites derived from *Alternaria* spp., tentoxin and tenuazonic acid showed the highest occurrence in TMRs, being detected in 79% and 53% of the samples, respectively. Tenuazonic acid presented the highest concentration in TMRs among this group of metabolites (average: 49.4 µg/kg, range: 30.3 µg/kg–82.8 µg/kg).

**Table 3:** Occurrences and concentrations of mycotoxins and other fungal metabolites detected in whole-plant corn silages and total mixed rations of Mexican dairy farms.

Group of Metabolites	Metabolite	Positive Samples ^1^ (%)	Whole-Plant Corn Silages (*n* = 19)			Positive Samples ^1^ (%)	Total Mixed Rations (*n* = 19)			Wilcoxon Matched-Pairs Test
			Concentration (µg/kg DM) ^2^				Concentration (µg/kg DM) ^2^			*p*-Value *
			Average ± SD	Median	Range		Average ± SD	Median	Range	
Ergot alkaloids	Festuclavine ^+^	5	–	–	2.41	0	–	–	–	>0.9999
	Dihydroergosine ^+^	26	1.35 ± 1.17	1.29	0.13–3.2	21	0.83 ± 0.97	0.44	0.18–2.28	0.0625
	Chanoclavine ^+^	5	–	–	2.04	5	–	–	12.5	>0.9999
*Alternaria* spp.	Altenuisol ^+^	32	2.5 ± 0	2.5	2.5–2.5	37	3.14 ± 1.7	2.5	2.5–6.99	0.7656
	Alternariol ^+^	5	–	–	5.5	11	9.77 ± 6.04	9.77	5.5–14	0.75
	Alternariolmethylether ^+^	47	9.89 ± 7.59	5.5	5.5–27.4	42	6.39 ± 2.51	5.5	5.5–12.6	0.25
	Altersetin ^+^	26	6.76 ± 4.5	5.16	1.25–12.7	42	15.7 ± 9.86	12.3	4.18–34.3	0.0488
	Infectopyron	21	97 ± 64	94	23.9–176	16	34.2 ± 3.38	36.1	30.3–36.2	0.1875
	Macrosporin ^+^	16	3.75 ± 0	3.75	3.75–3.75	11	3.75 ± 0	3.75	3.75–3.75	>0.9999
	Tentoxin ^+^	42	7.71 ± 4.86	6.38	3.1–16	79	6.91 ± 2.87	6.41	2.48–11.3	0.0932
	Tenuazonic acid ^+^	32	40.2 ± 10.4	37.5	30.1–60.4	53	49.4 ± 16.8	41.8	30.3–82.8	0.064
*Aspergillus* spp.	Averufin ^+^	42	3.6 ± 1.9	3.0	3.0–8.4	26	2.95 ± 0	2.95	2.95–2.95	0.125
	Deoxygerfelin	0	–	–	–	11	2.41 ± 1.33	2.41	1.47–3.35	0.5
	Flavoglaucin ^+^	11	2.8 ± 0.97	2.8	2.11–3.49	100	40.7 ± 29.6	41.6	3.63–111	<0.0001
	Fumigaclavine C ^+^	5	–	–	47.2	0	–	–	–	>0.9999
	Fumiquinazolin D ^+^	0	–	–	–	11	11.8 ± 5.34	11.8	8.01–15.6	0.5
	Kojic acid ^+^	11	877 ± 130	877	785–69	5	–	–	145	0.5
	Kotanin A	11	2.5 ± 0	2.5	2.5–2.5	5	–	–	2.50	>0.9999
	Methylsulochrin	5	–	–	4.5	11	4.5 ± 0	4.5	4.5–4.5	>0.9999
	Phenopyrrozin	84	56.1 ± 28.1	53.1	16.2–132	79	12.4 ± 5.14	10.7	7.16–24.1	<0.0001
	seco-Sterigmatocystin ^+^	16	2.72 ± 1.8	3.58	0.65–3.91	42	0.9 ± 0.46	0.65	0.65–1.71	>0.9999
	Sterigmatocystin ^+^	0	–		–	11	2.65 ± 0	2.65	2.65–2.65	0.5
	Versicolorin C	16	6.05 ± 3.98	3.75	3.75–10.6	0	–	–	–	0.25
*Fusarium* spp.	15-Acetyldeoxynivalenol ^+^	11	142 ± 46.7	142	109–175	0	–	–	–	0.5
	Fungerin	0	–	–	–	5	–	–	26.5	>0.9999
	Fusaproliferin ^+^	37	403 ± 628	166	61.5–1820	58	280 ± 252	226	60.8–989	0.3054
	Fusapyron ^+^	5	–	–	1.5	5	–	–	5.46–5.46	>0.9999
	Fusaric acid ^+^	89	1210 ± 840	1130	260–3220	74	562 ± 235	503	298–1190	<0.0001
	Hydrolysed Fumonisin B1 ^+^	16	37 ± 49.1	10	7.29–93.7	5	–	–	30.4	0.75
	Moniliformin ^+^	89	88.9 ± 76.9	48	9–263	100	101 ± 67	78.8	27.6–247	0.1956
	Nivalenol ^+^	42	269 ± 184	209	103–614	68	872 ± 853	385	88.5–2600	0.0061
	Sambutoxin ^+^	37	0.37 ± 0.19	0.3	0.3–0.79	5	–	–	0.3	0.0625
	Siccanol ^+^	89	4620 ± 3530	3960	525–12,350	95	2510 ± 1650	2370	409–6130	0.0028
	W493	79	171 ± 190	80.7	3.55–694	74	86.6 ± 65.4	101	3.55–190	0.0256
	Zearalenone ^+^	68	58.7 ± 79.4	21.5	4.6–278	100	38.7 ± 57.2	17.8	4.6–246	0.9297
	Total enniatins	47	5.96 ± 7.24	1.60	0.60–19	89	11.2 ± 9.8	7.11	1.85–37	0.0144
	Total fumonisins	47	1150 ± 1570	203	26.5–4410	89	325 ± 396	155	3.6–1670	0.3867
	Total Type B trichothecenes	53	2000 ± 1230	1790	323–4230	89	1940 ± 1760	1156	78.0–5510	0.0505
*Penicillium* spp.	7-Hydroxypestalotin	53	17.3 ± 9.99	14.9	7.3–41.9	47	9.74 ± 4.87	9.74	2.6–16.7	0.0186
	Asterric acid	5	–	–	12.5	5	–	–	12.5	N/A
	Bilaid A	100	20.3 ± 22.9	11.4	5.78–87.6	95	8.53 ± 7.04	6.77	3.49–27.3	<0.0001
	Citreoviridin ^+^	0	–	–	–	21	42.9 ± 12.2	41.3	31.1–58	0.125
	Citrinin ^+^	0	–	–	–	5	–	–	77.9	>0.9999
	Cycloaspeptide A	0	–	–	–	5	–	–	13.4	>0.9999
	Cyclopenin	5	–	–	2.85	0	–	–	–	>0.9999
	Mycophenolic acid ^+^	11	90.2 ± 118	90.2	7–173	42	32 ± 42.9	11.4	7–127	0.1094
	Mycophenolic acid IV ^+^	5	–	–	2.53	0	–	–	–	>0.9999
	NP 1243	5	–	–	34.1	0	–	–	–	>0.9999
	Oxaline	16	68.9 ± 61.2	81.2	2.55–123	16	20.1 ± 14.9	12.9	10–37.2	0.5
	Pestalotin	53	29.2 ± 13.5	28.7	8.61–59.2	58	12.4 ± 6.93	11.2	3.3–24.5	0.0282
	PF 1163A	5	–	–	3.32	5	–	–	0.75	>0.9999
	Questiomycin	5	–	–	1.5	89	8.71 ± 7.72	8.6	0.6–23	<0.0001
	Questiomycin Derivate	95	184 ± 111	164	34.9–407	95	118 ± 64.2	106	18.1–238	0.0002
	Quinolactacin A	11	1.2 ± 0	1.2	1.2–1.2	21	1.2 ± 0	1.2	1.2–1.2	0.5
Other fungi	Ascochlorin	21	11.9 ± 10.2	8.43	3.75–26.9	21	6.24 ± 4.99	3.75	3.75–13.7	0.625
	Ascofuranone	21	2.26 ± 1.82	1.35	1.35–4.98	5	–	–	1.35	0.3125
	Bassianolide	37	3.17 ± 1.25	2.7	2.7–6	32	2.7 ± 0	2.7	2.7–2.7	0.5
	Beauveriolide I_III	26	1.5 ± 0	1.5	1.5–1.5	16	4.22 ± 3.01	3.71	1.5–7.45	0.6563
	Cercosporin	58	40.8 ± 25.6	36.5	13.2–87.9	79	72.2 ± 79.7	42.5	15.1–325	0.0479
	Cytochalasin J	0	–	–	–	11	136 ± 26.5	136	117–155	0.5
	Destruxin B ^+^	0	–	–	–	21	1.25 ± 0.68	1.1	0.7–2.09	0.125
	Ilicicolin A	5	–	–	6.23	37	1.83 ± 0.61	1.6	1.6–3.21	0.2813
	Ilicicolin B	79	18.9 ± 20.7	4.45	4.45–69.4	89	14.1 ± 9.96	13	4.45–28.9	0.6848
	Ilicicolin E	5	–	–	1.7	11	1.7 ± 0	1.7	1.7–1.7	>0.9999
	Monocerin	89	115 ± 237	37.4	2.1–990	74	85.9 ± 133	37.2	2.1–502	0.0024
	Mycousnine	0	–	–	–	11	0.75 ± 0	0.75	0.75–0.75	0.5
	Myriocin ^+^	16	67.9 ± 52.1	48.1	28.6–127	32	44.6 ± 26.2	41.1	15.7–92.6	0.5625
	Phomalone	5	–	–	6.14	0	–	–	–	>0.9999
	Sporidesmolide II	84	7.9 ± 13.2	2.92	0.75–44.7	74	4.4 ± 5.07	2.54	0.75–17.2	0.0643
	Sporidesmolide III	5	0.75	0.75	0.75	0	–	–	–	>0.9999
Unspecific metabolites	3-Nitropropionic acid	21	63 ± 60.9	43	18.5–147	21	18.5 ± 0	18.5	18.5–18.5	0.5
	Asperglaucide	5	–	–	5.99	100	27.3 ± 33.6	10.8	2.05–142	<0.0001
	Asperphenamate	5	–	–	4.89	79	5.98 ± 7.37	3.35	1.93–31.4	<0.0001
	Brevianamid F	89	171 ± 77.8	166	61–408	89	116 ± 40.6	112	49.2–228	0.0021
	Chrysophanol	47	226 ± 111	231	62.5–367	32	176 ± 65.1	205	62.5–226	0.0195
	Citreorosein	53	24.1 ± 12.4	19.1	14.7–54.4	37	19.1 ± 6.67	15.8	12.5–30.1	0.123
	Cyclo(L-Pro-L-Tyr)	100	4680 ± 2300	4570	926–8970	100	2180 ± 1110	1890	589–5360	0.0006
	Cyclo(L-Pro-L-Val)	100	14,760 ± 3820	13,450	6890–2200	100	7080 ± 2300	6790	2160–11,570	<0.0001
	Emodin	95	9.62 ± 5.31	9.22	3.5–23.1	95	46.9 ± 102	8.49	3.5–422	0.2312
	Fellutanine A	95	128 ± 51.8	127	48.8–260	89	94.3 ± 38.7	86.7	34.8–199	0.0053
	Iso-Rhodoptilometrin	58	1.58 ± 0.59	1.4	1.4–3.35	53	1.4 ± 0	1.4	1.4–1.4	0.5
	N-Benzoyl-Phenylalanine	0	–	–	–	21	12.2 ± 2.47	12.1	9.56–15	0.125
	Neoechinulin A	0	–	–	–	100	133 ± 78.4	102	29.6–304	<0.0001
	Norlichexanthone	5	–	–	1.9	47	1.9 ± 0	1.9	1.9	0.0078
	Rugulusovine	100	355 ± 153	373	137–681	100	204 ± 93.8	197	53.5–407	<0.0001
	Skyrin	68	2.06 ± 1.07	1.85	0.55–3.96	89	4.42 ± 6.13	2.48	0.55–27	0.0097
	Ternatin	5	–	–	6.32	0	–	–	–	>0.9999
	Tryptophol	42	258 ± 126	170	170–456	32	963 ± 817	642	170–2100	0.5508
Phytoestrogens	Biochanin	5	–	–	147	79	36.3 ± 13	34.5	20.2–61.6	0.0081
	Coumestrol	26	56 ± 104	8	8–241	89	157 ± 126	109	45.5–479	0.0011
	Daidzein	37	263 ± 351	89	89–1020	100	12,700 ± 6710	10,710	3820–27,620	<0.0001
	Daidzin	68	428 ± 719	191	91–2730	100	63,690 ± 40,170	65,640	9350–125,770	<0.0001
	Genistein	58	153 ± 272	47	47–947	100	11,760 ± 6170	11,190	3;990–26,530	<0.0001
	Genistin	63	1000 ± 1850	362	110–6700	100	118,150 ± 75,850	113,270	157,180–249,320	<0.0001
	Glycitein	5	–	–	324	89	4790 ± 1840	4450	2220–8220	<0.0001
	Glycitin	11	364 ± 292	364	158–570	100	13,340 ± 7920	12,070	1080–27,390	<0.0001
	Ononin	5	–	–	46	100	176 ± 28	153.3	46–512	<0.0001
Other plant metabolites	Abscisic acid	42	1610 ± 2860	574	273–8670	100	1660 ± 636	1620	411–3270	0.0012
	Anisodamine	16	514 ± 373	470	164–907	16	137.2 ± 101	141	34.5–236	0.375
	Atropine	16	318 ± 85	360	219–374	11	69.1 ± 22.4	69.1	53.3–84.9	0.25
	Hyoscine	16	427 ± 391	473	15–794	11	215.7 ± 93.1	216	150–282	0.375
Bacterial	Nonactin	16	1 ± 0	1	1–1	26	1.3 ± 1.2	0.8	0.6–3.3	0.3906

^1^ Samples with values > limit of detection (LOD). ^2^ Excluding data < LOD. In case values > LOD and <limit of quantification (LOQ), LOQ/2 was used for calculation. * Significant differences between each set of matched pairs presented *p*-value < 0.05. SD = Standard deviation; DM = Dry matter; ^+^ = metabolites classified as mycotoxins.

Tenuazonic acid presented the highest concentration in TMRs among this group of metabolites (average: 49.4 µg/kg, range: 30.3 µg/kg–82.8 µg/kg). In the analyzed silages, the most frequently occurring were alternariolmethylether (47%) and tentoxin (42%). The *Alternaria*-derived metabolites with the highest levels were infectopyrone (average: 97 µg/kg; range: 23.9 µg/kg–176 µg/kg) and tenuazonic acid (average: 40.2 µg/kg; range: 30.1 µg/kg–60.4 µg/kg). Other metabolites produced by *Alternaria* spp. such as altenuisol, alternariol, altersetin, and macrosporin were also detected in both studied matrices. Among the metabolites produced by fungi of the genus *Aspergillus*, flavoglaucin was detected in all the TMR samples, followed by phenopyrrozin, which was found in 79% of the samples. Phenopyrrozin also showed the highest occurrence of Aspergilli-derived compounds in WPCSs. Kojic acid presented the highest levels among the *Aspergillus*-derived metabolites in silages and TMRs, although its occurrence was low. Other metabolites/mycotoxins such as averufin, fumigaclavine, fumiquinazolin D, and seco-Sterigmatocystin were detected in both WPCSs and TMRs. Among the ergot alkaloids, dihydroergosine occurred the most. Chanoclavine was detected in low frequency (5%) in TMRs and WPCSs. Festuclavine was detected only in WPCSs. The concentrations of the individual ergot alkaloids were low (≤12.5 µg/kg). Concerning the compounds produced by other fungi species, the most frequently detected in TMR samples were ilicicolin B (89%), cercosporin (79%), monocerin (74%), and sporidesmolide II (74%). The metabolites derived from other fungal species that were most frequently detected in the analyzed WPCS samples were monocerin (89%), sporidesmolide II (84%), and ilicicolin B (79%). Monocerin was the compound derived from other fungi with the highest concentration in WPCSs as well as in TMRs, with a respective average of 115 µg/kg and 85.9 µg/kg. The levels of monocerin were significantly superior (*p*-value = 0.0024) in the silages. Samples of silage and TMRs showed content of ascochlorin, ascofuranone, bassianolide, beauveriolide III, ilicicolin A, and myriocin. Cytochalasin J, destruxin B, and mycousnine were identified only in TMRs, whereas phomalone and sporidesmolide III were identified only in WPCSs.

#### 2.2.3. Plant Secondary Metabolites (Phytoestrogens and Others)

Among the plant-derived metabolites, nine phytoestrogens in WPCSs and in TMRs were found. The isoflavones daidzein, daidzin, genistein, genistin, and glycitin and the isoflavone glucoside onionin were identified in all the TMR samples. Biochanin, coumestrol, and glycitein also presented a high occurrence in TMRs (≥79%). The most frequently detected phytoestrogens in WCPSs were daidzin (68%), genistin (63%), and genistein (58%). The other phytoestrogens were identified in <40% of the silage samples. The concentrations of the all the found phytoestrogens were significantly higher (*p*-value < 0.5) in the TMRs than in the WPCSs. Genistin was the (plant) metabolite with the highest concentration detected in both feed matrices, averaging 1000 µg/kg and 118,150 µg/kg in WPCSs and TMRs, respectively. Concerning the compounds cataloged in the group of other plant metabolites, abscisic acid was predominant in occurrence and concentration; for instance, its occurrences in WPCSs and TMRs were 42% and 100%, respectively. The levels of abscisic acid were significantly superior (*p*-value = 0.0012) in TMRs than in the silages. Three tropane alkaloids (anisodamine, atropine, and hyoscine) were identified in both analyzed matrices. The 3 mentioned tropane alkaloids occurred in 16% of the WPCSs, whereas in TMRs anisodamine was found at a frequency of 16% and atropine as well as hyoscine in 11% of the samples. These alkaloids were detected in concentrations lower than 1000 µg/kg; the levels were higher in WPCSs, but without significance (i.e., *p*-value > 0.05) (Table 3).

#### 2.2.4. Unspecific (Multi-Kingdom) and Bacterial Metabolites

Multiple metabolites that can be produced by unrelated organisms belonging to diverse kingdoms such as Plantae, Fungi, and/or Eubacteria were detected in both feed commodities. Four compounds belonging to this category, asperglaucide, cyclo (L-Pro-L-Tyr), cyclo (L-Pro-L-Val), neoechinulin A, and rugulusovine, were identified in all the assessed TMR samples. Asperphenamate, brevianamid F, chrysophanol, emodin, skyrin, fellutanine A, and iso-rhodoptilometrin occurred in TMRs at a rate superior to 50%. Other unspecific metabolites such as 3-nitropropionic acid, chrysophanol, citreorosein, N-benzoyl-phenylalanine, and tryptophol were detected in frequencies between 20% and 40%. Regarding the occurrence in WPCSs, all the assessed samples contained cyclo (L-Pro-L-Tyr), cyclo (L-Pro-L-Val), and rugulusovine, with frequencies of over 50% for brevianamid F, citreorosein, emodin, fellutanine A, iso-rhodoptilometrin, and skyrin. The highest concentrations in the category of unspecific metabolites corresponded to the bioactive cyclic dipeptides cyclo (L-Pro-L-Val) and cyclo (L-Pro-L-Tyr) in silages as well as in TMRs, with an average concentration above 2100 µg/kg. The content of both cyclic dipeptides was significantly higher (*p*-value < 0.001) in WCPSs than in the complete rations. The other compounds of this group presented average concentrations lower than 400 µg/kg in WPCSs and TMRs (Table 3).

### 2.3. Co-occurrence of Mycotoxins and Phytoestrogens

**Figure 2:**
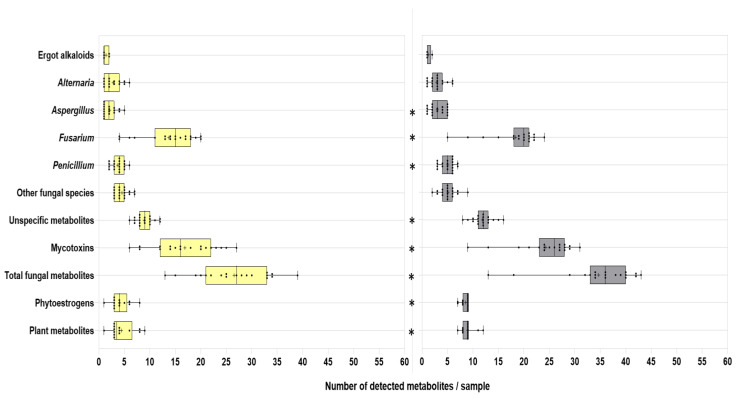
Scatter plots illustrate the grade of co-contamination (number of metabolites/sample) by group, whole-plant corn silages (in yellow) or total mixed rations (in gray) from Mexico. Asterisks (*) confirm significant differences (*p*-value < 0.05) between the number of metabolites per sample in the respective group, whole-plant corn silages or total mixed rations, according to the Wilcoxon matched-pairs signed rank test (*p*-values in Table S1).

Figure 2 illustrates the distribution and variation grade of the individual samples in the co-contamination levels of the metabolite groups. Table S1 presents the exact values of the co-contamination levels (average ± SD, median, minimum, maximum) of the groups of metabolites. Additionally, the significance (*p*-values) of the comparison via the Wilcoxon matched-pairs signed rank test between co-contamination of the diverse groups’ metabolites in samples of WPCSs and TMRs are also presented in Table S1. All the samples were co-contaminated with cocktails of toxins/metabolites. In total, the assessed WPCSs showed, on average, 29 metabolites per sample (range: 13–39 metabolites per sample), and TMRs showed 55 metabolites per sample (range: 31–66 metabolites per sample). Silages presented, on average, 17 mycotoxins per sample, varying from 6 to 27 mycotoxins per sample. The assessed TMR samples showed a mean of 24 mycotoxins per sample, ranging from 9 to 31 mycotoxins per sample. The mycotoxin co-contamination level was significantly higher (*p* < 0.0001) in TMRs. *Fusarium*-derived metabolites was the category among the fungal metabolites with the highest diversity of detected compounds, showing, on average, 14 metabolites per sample (max: 20 metabolites per sample) in WPCSs and 18 metabolites per sample (max: 24 metabolites per sample) in TMRs. The co-contamination grade with compounds derived from *Aspergillus* spp., *Fusarium* spp., *Penicillium* spp., and other fungi, in addition to the total fungal metabolites, phytoestrogens, plant metabolites, unspecific metabolites, and total metabolites, was significantly higher (*p*-value < 0.05) in the complete rations than in the silages.

**Figure 3:**
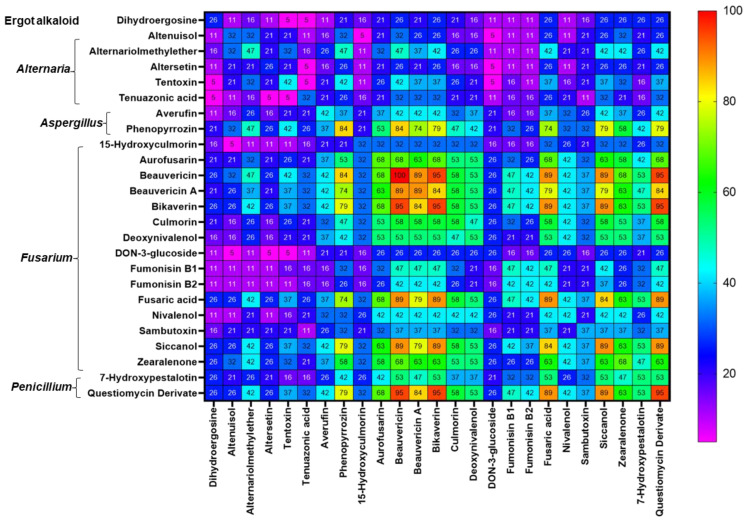
Heatmap of the most frequent combinations of mycotoxins/metabolites detected in whole-plant corn silages from Central and Northern Mexico dairy farms. Values correspond to percentage of samples containing both mycotoxins/metabolites. Mycotoxins/metabolites included in this analysis occurred in ≥25% of the samples.

**Figure 4:**
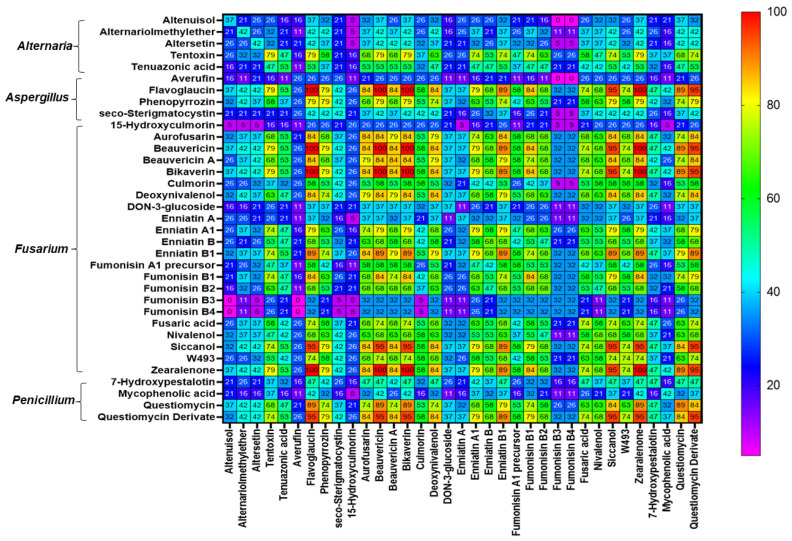
Heatmap of the most frequent combinations of mycotoxins/metabolites detected in total mixed rations from Central and Northern Mexico dairy farms. Values correspond to percentage of samples containing both mycotoxins/metabolites. Mycotoxins/metabolites included in this analysis occurred in ≥25% of the samples.

Co-occurrence analyses (frequency of detection of combinations, in %) between mycotoxins/metabolites evidenced in WPCSs and TMRs are presented in Figure 3 and Figure 4, respectively. In the silages, the most frequent combinations of mycotoxins detected were among ZEN and fusarial emerging mycotoxins such as aurofusarin, beauvericin, beauvericin A, bikaverin, fusaric acid, and siccanol, which presented occurrences of over 50%. The co-occurrence of DON and ZEN was detected in 53% of the WPCS samples. The *Aspergillus*-produced metabolite phenopyrrozin and the *Penicillium*-derived questiomycin derivate showed co-occurrences of over 50% with ZEN and the emerging fusarial mycotoxins such as aurofusarin, beauvericin, beauvericin A, bikaverin, fusaric acid, and siccanol (Figure 3). Regarding the most recurrent combinations of mycotoxins/metabolites in TMRs, the co-occurrence among aurofusarin, beauvericin, beauvericin A, bikaverin, fusaric acid, and siccanol were higher than 75%. The mycoestrogen ZEN presented a high degree of co-occurrence with mycotoxins DON (84%), FB1 (84%), FB2 (68%), and NIV (68%). Remarkably, more than half of the TMR samples presented co-occurrence of the *Alternaria*-derived mycotoxins alternariolmethylether and tenuazonic acid and the *Aspergillus*-produced flavoglaucin and phenopyrrozin; questiomycin and its derivate also co-occurred with the fusarial mycotoxins DON, ZEN, FB1 aurofusarin, beauvericin, beauvericin A, bikaverin, and enniatin B1 (Figure 4).

**Figure 5:**
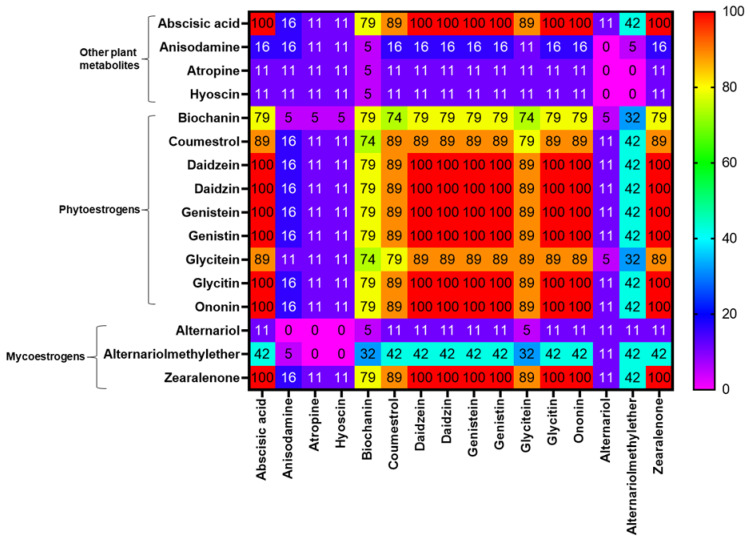
Heatmap of the combinations of detected plant metabolites (including phytoestrogens) and mycoestrogens in total mixed rations from Central and Northern Mexico dairy farms. Values correspond to percentage of samples containing both metabolites.

The co-occurrence rates of estrogenic compounds (for instance, phytoestrogens and mycoestrogens) and other detected plant secondary metabolites (abscisic acid, anisodamine, atropine, and hyoscine) in TMRs are shown in Figure 5. All tested samples presented co-occurrence among the phytoestrogens daidzein, daidzin, genistein, genistin, glycitin, and onionin and the fusarial mycoestrogen ZEN. The occurrence of these mentioned estrogenic compounds with the *Alternaria* mycoestrogens alternariol and its monomethylether corresponded to 11% and 42%, respectively.

### 2.4. Relationship between Concentrations of Mycotoxin/Metabolite Groups and the Dietary Ingredients

**Figure 6:**
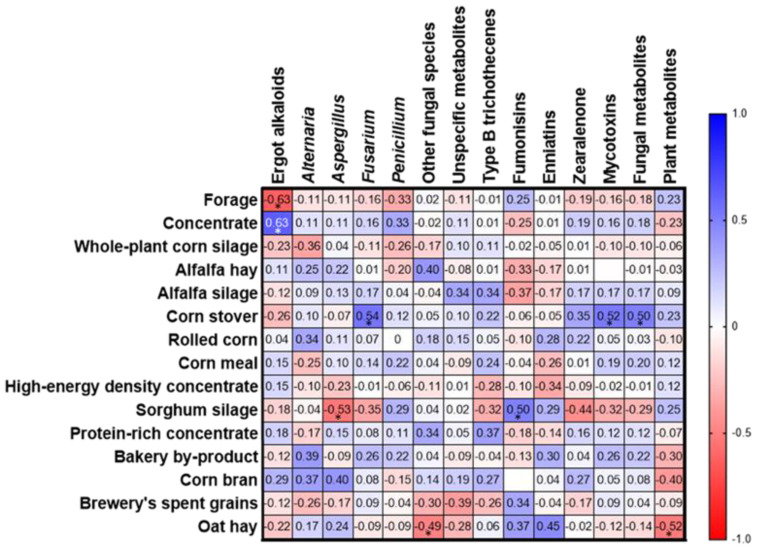
Spearman’s correlation coefficients (*ρ*) among groups of metabolites detected in total mixed rations with the main ingredients. The asterisks (*) indicate significant correlation coefficients (*p*-value < 0.05). All the *p*-values are available in Table S2.

Spearman’s correlation coefficients (rho(*ρ*)) among groups of metabolites detected in total mixed rations with the main ingredients of the TMRs are shown in Figure 6. The respective *p*-values of the correlation coefficients are presented in the supplementary Table S2. Correlations with individual mycotoxin, phytoestrogen, and tropane alkaloid levels were also assessed (data not shown); strong and moderate correlation coefficients and their respective *p*-values are presented in the text. A moderate positive correlation was observed between the total proportion of concentrate with total ergot alkaloids (*ρ* = 0.63, *p*-value = 0.038), the ergot alkaloid dihydroergosine (*ρ* = 0.63, *p*-value = 0.006), and the *penicillium*-derived metabolite pestalotin (*ρ* = 0.59, *p*-value = 0.008). The dietary content of corn stover presented a moderate positive correlation with levels of *Fusarium*-derived metabolites (*ρ* = 0.63, *p*-value = 0.0173), beauvericin (*ρ* = 0.60, *p*-value = 0.007), total mycotoxins (*ρ* = 0.52, *p*-value = 0.0220), and total fungal metabolites (*ρ* = 0.50, *p*-value = 0.0305). Sorghum silage showed a moderate positive correlation with the total Fs levels (ρ = 0.65, *p*-value = 0.0303), FA2 (*ρ* = 0.65, *p*-value = 0.0028), FB2 (*ρ* = 0.50, *p*-value = 0.0275), FB3 (*ρ* = 0.52, *p*-value = 0.0213), FB4 (*ρ* = 0.57, *p*-value = 0.0109), hydrolyzed FB1 (*ρ* = 0.73, *p*-value = 0.0004), and citrinin (*ρ* = 0.73, *p*-value = 0.0004). The dietary content of oat hay revealed a moderate positive correlation with the concentration of the *Fusarium* emerging mycotoxin enniatin B2 (*ρ* = 0.68, *p*-value = 0.0012) and the tropane alkaloid anisodamine (*ρ* = 0.52, *p*-value = 0.0236) and negative correlation with the metabolites produced by other fungi (*ρ* = 0.52, *p*-value = 0.0331) and total plant metabolites/phytoestrogens (*ρ* = 0.52, *p*-value = 0.0231). Brewery spent grains correlated positively and moderately with the alkaloid tropanes anisodamine (*ρ* = 0.54, *p*-value = 0.0165), atropine (*ρ* = 0.65, *p*-value = 0.0028), and hyoscine (*ρ* = 0.65, *p*-value = 0.0028).

## 3. Discussion

This investigation describes for the first time the occurrence of mixtures of mycotoxins, phytoestrogens, and other secondary metabolites in the WPCSs and TMRs of dairy farms in Mexico. The presented results confirmed the ubiquitous presence of mycotoxin mixtures in feeds and complete rations of dairy cows, as indicated in previous studies [2,22,39,40,41]. The multi-mycotoxin approach used in this study showed that previous reports on mycotoxin contamination are underestimations, as demonstrated by the mixtures fluctuating from 9 to 31 different mycotoxins (13 to 43 total fungal metabolites and 31 to 66 total secondary metabolites) per ration, evidencing the realistic scenario of simultaneous dietary exposition of dairy cattle to multiple mycotoxins and endocrine disruptors. *Fusarium*-derived mycotoxins/metabolites represented the most relevant fungal metabolites considering the high co-occurrence rates and levels in both WPCSs and TMRs. Our outcome confirms the importance of *Fusarium* spp. as a primary contributor to contamination with mycotoxins (such as ZEN, DON, and Fs), emerging mycotoxins (such as beauvericin), and other less studied metabolites in dairy cattle feeds, which have also been described in other regions such as South America [42], Europe [2,5,43], and Asia [22].

Mexican regulations establish maximum limits only for AFs in cereals and cereal products. The maximal levels for AFs for humans are 20 µg/kg and for cattle consumption 100–300 µg/kg [44,45]. No limits or guidance levels are set for other mycotoxins. Because Mexican regulations on mycotoxins in animal feed are outdated and not strict enough, they should be updated accordingly [46]. As reference values for this discussion, the advisory limits/guidance values established by the FDA and EU Commission [19,47,48] will be considered. On an 88% DM basis, the FDA sets for complete rations of dairy animals levels of 5000 µg DON/kg, 30,000 µg Fs (FB1 + FB2 + FB3)/kg, and 20 µg AFs/kg. For OTA (and others ochratoxins) as well as ZEN, the FDA has no regulatory limits [47,48]. The EU Commission recommends 500 µg/kg for ZEN and 5000 µg/kg for DON [19]. Previous studies on mycotoxins in Mexico cattle feeds focused mainly on classic mycotoxins such as AFs, Fs, OTs, ZEN, and DON [11,49]. In the case of the individual mycotoxin levels such as DON (max: 1670 µg/kg), ZEN (max: 246 µg/kg), and total Fs (max: 1670 µg/kg), no sample presented contamination levels higher than the FDA or EU Commission’s regulatory levels. However, the sum of related toxic metabolites can be higher than such regulatory limits, for example, the sum type B trichothecenes (amount of DON, 15-acetyl-DON, DON-3-glucoside, and NIV). The highest concentration of total type B trichothecenes detected in TMR samples was 5510 µg/kg (equivalent to 6405 µg/kg on an 88% DM), which is above the levels of the single parent mycotoxin included in the legislation (DON). This evidence shows that although the analysis of individual analytes can be below the guidance levels, the total content of related metabolites such as modified mycotoxins, in this case type B trichothecenes, can be above the guidance value, representing a risk. Our results confirm that mycotoxin regulations target the tip of the iceberg if we consider the multiple mycotoxins co-occurrence (i.e., the real-world scenario), as suggested previously [2,4]. In fact, common combinations of mycotoxins detected in the TMR samples from Mexico such as DON and ZEN (84%), ZEN and NIV (68%), ZEN and FB1 (84%), and NIV and DON (63%) present synergistic effects [50,51,52,53,54]. McKay et al., 2019 demonstrated that diets (TMR) contaminated with concentrations under the FDA or EU Commission guidance levels, for instance, 1966 μg DON/kg DM and 366 μg ZON/kg DM, declined milk yield to 0.74 kg/cow/d, which can reduce the income of farmers significantly [55].

TMRs of Mexican dairy cows also were contaminated with other less studied compounds derived from *Fusarium* (such as beauvericin, enniatins, culmorin, and bikaverin), *Alternaria* (e.g., alternariolmethyether and teanuazonic acid), and *Aspergillus* (e.g., kojic acid, averufin, and STC) along with *Penicillium* toxins (such as mycophenolic acid) and other metabolites that have been reported previously in the diets and silages of dairy cattle [2,22,43,56]. As in preceding studies, the current research found *Fusarium* mycotoxins/metabolites to be a dominant group of fungal metabolites in WPCSs and TMRs [2,43]. The occurrences and average levels of enniatins and ergot alkaloids were lower than those of diets and maize silages in Europe reported recently [2,43], which suggests that these kinds of toxins occur less in Mexico, representing a lower risk than in Europe. However, this must be confirmed by additional studies. On the other hand, the occurrence and total Fs levels were higher in the TMRs of Mexican dairy farms (occurring in 89% of the samples; average: 325 µg/kg; max: 1670 µg/kg) than the dietary levels of total Fs previously reported in Austria (with an occurrence of 71%; average: 150 µg/kg; max: 1590 µg/kg) [2]. The occurrence of FB1 (34.8%) and FB2 (29.1%) in a European survey on mycotoxins in WPCSs [43] was lower than that evidenced in our study (FB1: 47%; FB2: 42%). Comparing the median and maximum concentrations of Fs in WPCSs from Mexico showed averages (FB1 median: 124 µg/kg; max: 2703 µg/kg; FB2 median: 301 µg/kg; max: 987 µg/kg) higher than the averages of European WPCSs (FB1 median: 60 µg/kg; max: 553 µg/kg; FB2 median: 20.4 µg/kg; max: 133 µg/kg) [43]. The same trend was evidenced for DON occurrences and levels in Mexican (100%; median: 1370 µg/kg; max: 3352 µg/kg) and European (67.7%; median: 303 µg/kg; max: 3060 µg/kg) WPSCs. This study did not find samples contaminated with OTA, a mycotoxin with low occurrence in European WPCSs (2.5%) [43] and Austrian complete rations (1%).

ZEN also presented higher occurrence and median in Mexican (100%; 15.2 µg/kg) than in European (67.7%; µg/kg) WPSCs. However, the ZEN maximum level detected in a WPCS sample from Europe (1670 µg/kg) was around 6 times higher than that reported in the present study (278 µg/kg). Although our investigation did not detect AFB1 and OTA, as initially expected, it is essential to clarify that these fungal compounds with carcinogenic properties have been widely reported in dairy cattle feeds (such as cereals) and dairy products (AFM1) in Mexico, representing a real and latent veterinary and public health risk in this country [12,46,49,57,58,59,60]. Averufin, sterigmatocystin, and versicolorin C, considered to be possible precursors of AFs [61,62,63,64,65], were detected. Sterigmatocystin was previously reported in Mexican maize [59]. Like AFs, sterigmatocystin is known to be a carcinogenic compound with immunotoxin and immunomodulatory activity. Data on the exposure of dairy cows and other animals to sterigmatocystin and the related toxicological implications are limited [66,67,68].

Our results highlight teanuazonic acid as one of the most abundant *Alternaria* mycotoxins in TMRs. However, animal epidemiological and toxicological information on *Alternaria*-produced toxins (e.g., alternariol, alternariolmethylether, and teanuazonic acid) is still required. Health risks associated with *Alternaria* toxins in feeds must be investigated and clarified [69]. We also detected the *Penicillium*-derived compound mycophenolic acid, mainly related to post-harvest contamination during the ensiling process [8,40,70]. Previous studies showed average levels of mycophenolic acid of 54 µg/kg and 47.5 µg/kg in TMRs from the Netherlands and Austria, respectively [2,71]. The mean of the evaluated TMR samples (32 µg/kg) was lower than the cited European reports. Additionally, kojic acid, produced primarily by *Aspergillus* spp. but also by some *Penicillium* spp. [72], has demonstrated antibacterial and immunomodulatory activity [73,74,75]. Citrinin, which is primarily *Penicillium*-derived [76] but also produced by some *Aspergillus* spp. [77], was also found. Moreover, several less known metabolites produced by other fungi were detected in TMRs. Some of them, such as cercosporin, the illicicolins, and cytochalasins, have antibacterial activity [78,79,80,81]. The diversity of mycotoxins and fungal secondary metabolites detected in TMRs is due to their multi-commodity composition (Table 1).

Concerning the risk associated with toxicological interactions of mycotoxins [4,82], this study demonstrated a high occurrence of a wide variety of mycotoxins (most of them not considered in legislation at the international level) and other fungal secondary metabolites in the TMRs of dairy cattle. In addition, our findings also showed that phytoestrogens constituent a class of metabolites ubiquitously contained in dairy cow rations. The concern in veterinary medicine and public health related to phytoestrogens is due to their endocrine-disrupting activity. These estrogenic compounds are found primarily in Leguminosae plants, such as clovers (*Trifolium* spp.), alfalfa (*Medicago sativa*), and soybeans (*Glycine max*), and they can act as endocrine disruptors, impairing the reproductive performance of livestock [25,26,27,28,29,30,31,32,33,34,35,36,37,83,84]. In TMR samples, the phytoestrogens that most occurred and the highest concentrations presented were isoflavones such as genistin, daidzein, glycitin, and daidzein (Table 3). However, coumestrol, which is reported to be more potent in estrogenic activity than isoflavones, presented concentrations below the reported critical range (18–180 mg/kg) [85]. The interaction of phytoestrogens with other estrogenic xenobiotics (such as mycoestrogens) is currently the focus of interest [86,87,88]. In this study the co-occurrence of these estrogenic compounds with mycoestrogens such as ZEN, alternariol, and alternariolmethyether was corroborated in TMRs of dairy cattle (Figure 5), matching previous results of a similar survey carried out in Austria [2]. Along with the mentioned phytoestrogens, other plant-derived compounds detected in TMRs of dairy cows were the phytohormone abscisic acid [89] and the tropane alkaloids anisodamine, atropine, and hyoscine [90]. These alkaloids can have a wide range of biological activity (e.g., anticholinergic effects) and are mostly detected in high concentrations in plants belonging to the Solanaceae and Erythroxylaceae families [91]. These tropane alkaloids were previously detected in cattle feed from Tunisia and Spain in lower concentrations [92] than those presented here. However, according to a scientific opinion of the panel on contaminants of the European Food Safety Authority (EFSA), toxicosis due to tropane alkaloids in livestock is relatively rare [93]. We consider the presence of these alkaloids in TMRs to be a consequence of the existence of native Solanaceae weeds in the feed crops of Mexican dairy cattle. Due to the detected occurrences (≤16%) and low concentrations (<300 µg/kg) in TMR samples, these alkaloids seem not be a risk for the fed cattle.

Our results revealed corn stover as the most correlated ingredient with the content of total mycotoxins, *Fusarium*-derived metabolites, and fungal metabolites (Figure 6). Corn stover is the stalks, leaves, and husks that remain in the field after corn harvest [94]. It has been reported as a source of abundant exposure to *Fusarium* mycotoxins such as Fs, ZEN, and DON [95,96]. The content of ergot alkaloids correlated to the proportion of concentrate in the diet, confirming previous reports that related cereal grains with ergot alkaloids [97]. The proportion of sorghum silage in the rations presented the highest correlation with total content of Fs but also of FA2, FB2, FB3, FB4 hydrolyzed FB1, and citrinin. A previous study performed in the state of Nuevo León, Mexico, evidenced a contamination rate by Fs of 62% [98]. In Uruguay, it was found that 40% of the freshly harvested samples of sorghum presented contamination with Fs [99]. In Brazil, the occurrence of FB1 in sorghum was 74% [100]. These reports demonstrated that Fs contamination is common in this crop. In contrast to prior investigations in other regions such as Europe and South America [2,26,43,71], our results do not suggest WPCSs as one of the most contributing feedstuffs to mycotoxin/metabolite contamination. Concerning the correlations between the dietary ingredients and the levels of mycotoxins/metabolites, it is crucial to consider that more consistent association and relationship assessments require higher sample sizes and additional studies.

The complex mixtures of different mycotoxins, phytoestrogens, and other metabolites evidenced in the WPCSs and rations of dairy cattle in Mexico indicate, along with previous reports/studies, that unexplored and unpredictable toxicological interactions, such as synergistic as well as antagonistic toxic effects, are happening. Extensive studies using a multi-metabolite approach should be performed in other Mexican regions and other Latin American countries on dairy feed and other animal feed but also food for human consumption, including animal-derived products such as dairy products. More governmental interest and research are essential to ensure the safety of animal feed and derived foods, which will support animal health and the productive potential of herds, as well as the delivery of safe products to consumers.

## 4. Conclusions

This study demonstrated the ubiquitous contamination of WPCSs and TMRs by a wide spectrum of mycotoxins/metabolites (derived from the genera *Fusarium*, *Alternaria*, *Aspergillus*, and *Penicillium*) and endocrine disruptor compounds such as phytoestrogens and other metabolites in Mexico. Overall, *Fusarium*-produced mycotoxins and metabolites were the dominant fungal contaminants. In the assessed TMR samples, ZEN was found with a frequency of 100%, Fs of 89%, and DON of 84%. Although the detected individual levels of the classic mycotoxins (ZEN, DON, FB1, and FB2) were below the maximum/guidance values of Mexican, EU, and FDA regulations, the fact that multiple (regulated, modified, and emerging) mycotoxins co-occurred in complex mixtures, fluctuating from 9 to 31 toxins per sample, should cause concern. Most detected mycotoxins/metabolites are not well studied; their effect as mixtures and their toxicological implications have not been determined. Long-term and subclinical effects on herds’ health, production, and reproduction produced by complex mixtures of toxins and endocrine disruptors are unpredictable and require more research. Regarding the ingredients that represent more risk for mycotoxin contamination in TMRs, corn stover was the most correlated feedstuff to high total mycotoxins levels, and sorghum silage was most correlated to Fs contamination. Our results also revealed that dietary concentrate proportion had the strongest correlation to ergot alkaloid contamination in the TMRs of Mexican dairy cattle.

## 5. Materials and Methods

### 5.1. Sampling and Sample Preparation

**Figure 7:**
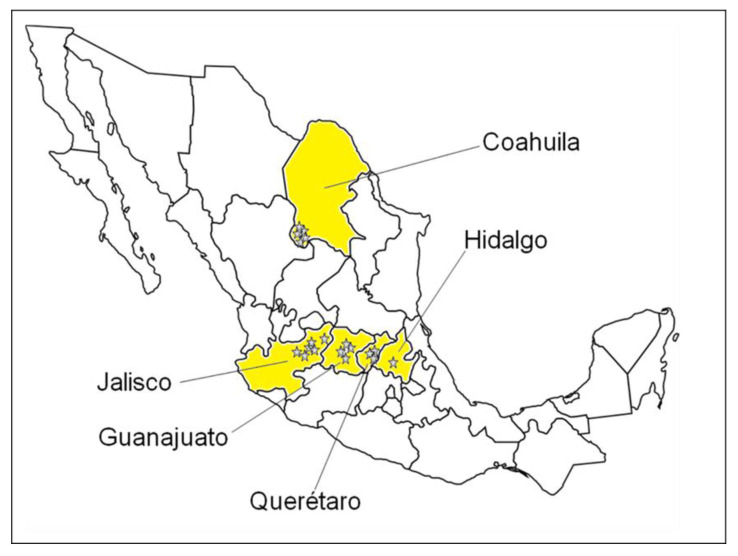
Map of Mexico showing the locations of the participating farms.

Representative samples of TMRs and WPCSs were collected from 19 dairy farms in 5 states in northern and central Mexico—for instance, Coahuila (5), Guanajuato (3), Hidalgo (1), Jalisco (7), and Querétaro (3) (Figure 7). The average herd size of the participating farms was 1512 (SD ± 986) lactating cows, varying from 100 to 3500 lactating cows. The main cattle breed of the farms was Holstein-Friesian. Each representative sample of TMRs and WPCSs consisted of at least of 30 incremental samples. Data on the TMR formulation (most important ingredients and their respective proportions) were collected via personal interview (questionnaire-guided).

The samples were manually collected with gloves from the feed bunk directly after the serving (TMR) (according to Penagos-Tabares et al., 2022 [2]) and from already-opened and “ready to be fed” WPCS bunker silos (according to McElhinney et al., 2016 [101]). The amount of composited samples (>30 incremental samples) was 1–1.5 kg. Collected samples were homogenized (properly manually mixed), vacuum-packed, and stored in the dark at −20 °C until sample preparation. Sampling was carried out during the period of July–August of 2022. For the sample preparation, TMR and WPCS samples were air-dried (at 65 °C for 48 h) and the whole samples were subsequently milled to a final particle size < 0.5 mm using a mill (Hamilton Beach Model 80335R, Hamilton Beach Brands Inc., China). Finally, aliquots of 5 grams (±0.01 g) of each homogenized representative sample were designed for analysis. The samples were placed into 50 mL polypropylene conical tubes (Sarstedt, Nümbrecht, Germany) and sent to Tulln an der Donau, Austria, for multi-metabolite analysis. The sample preparation was carried out at the Laboratory of Animal Nutrition of Facultad de Estudios Superiores Cuautitlán, Medicina Veterinaria y Zootecnia (UNAM), located in Cuautitlán Izcalli, México.

### 5.2. Multi-Mycotoxin Analysis (LC-ESI–MS/MS)

The validated multi-metabolite (>800) liquid chromatography/electrospray ionization–tandem mass spectrometric (LC/ESI–MS/MS) method was carried out at the Institute of Bioanalytics and Agro-Metabolomics of the University of Natural Resources and Life Sciences, Vienna, located in Tull an der Donau, Austria, according to previous descriptions. Water purification was completed using a Purelab Ultra system (ELGA LabWater, Celle, Germany). Glacial acetic acid (p.a.) and ammonium acetate (LC-MS grade) were bought from Sigma-Aldrich (Vienna, Austria). HiPerSolv Chromanorm HPLC gradient grade acetonitrile was purchased from VWR Chemicals (Vienna, Austria), and LC-MS Chromasolv grade methanol was acquired from Honeywell (Seelze, Germany). Standards of >800 fungal, plant, and unspecific secondary metabolites were supplied by several research institutions or commercial providers and are listed in Supplementary Table S3. For simultaneous quantification of multiple metabolites, 5 grams (±0.01 g) of each TMR and WPCS sample was extracted in 20 mL of the extraction solvent (acetonitrile/water/acetic acid 79:20:1, *v*/*v*/*v*) following the procedures reported by Steiner et al. (2020) [102]. These volumes were placed into the QTrap 5500 LC-MS/MS system (Applied Biosystems, Foster City, CA, USA) equipped with a TurboV electrospray ionization (ESI) source coupled to a 1290 series UHPLC system (Agilent Technologies, Waldbronn, Germany). Subsequently, quantification from external calibration by serial dilutions of a stock solution of analyzed compounds was accomplished. Finally, the outcomes were adjusted for apparent recoveries defined through spiking experiments, according to Steiner et al. (2020) [102]. This analytical methodology has been validated [96] and used to study the occurrence of multiple metabolites in complex feedstuff matrices such as silages, pastures, concentrates, and TMRs [2,5,22,39,56]. The method accuracy has been verified on a routine basis by proficiency testing organized by BIPEA (Genneviliers, France). Satisfactory z-scores between −2 and 2 have been achieved for >95% of >1800 results submitted so far. Supplementary Table S4 presents performance values of LC/ESI–MS/MS analysis for mycotoxins, phytoestrogens, and other fungal, plant, and unspecific metabolites detected in WPCSs and TMRs.

### 5.3. Data Analysis

Concentrations of metabolites were presented in μg/kg on a DM basis. Descriptive statistics (i.e., occurrences and the average, median, and range of the concentrations) were processed considering only the positive values (x ≥ limit of detection (LOD)) using Microsoft^®^ Excel^®^. Values lower than the limit of quantification (LOQ) were calculated as LOQ/2. The normality assessment of the data was completed via the D’Agostino and Pearson test, Anderson-Darling test, Shapiro-Wilk test, and Kolmogorov-Smirnov test. All the tests indicated the non-normal distribution of the handled data. Considering the dependence, the differences between concentrations of metabolites in TMRs and WPCSs of each respective farm were assessed via the (nonparametric) Wilcoxon matched-pairs signed rank test, and statistical differences were considered significant at *p*-value < 0.05. The co-occurrence analyses of mycotoxins and plant metabolites were performed separately using Microsoft Excel, generating matrices plotted in heatmaps. Moreover, a two-tailed Spearman’s correlation test was conducted to explore possible relations among dietary ingredients and levels of metabolites. Spearman’s correlation coefficients were considered significant at a *p*-value < 0.05. Accordingly, the correlation coefficients were interpreted according to Hinkle et al. 2003 [103]: “very high” (0.90 up to 1.00), “high” (0.70 up to 0.90), “moderate” (0.50 up to 0.70), “low” (0.30 up to 0.50), and “negligible” (<0.30). Low and negligible correlations were not considered for the interpretation of the results. The statistical analyses and graphs were completed using GraphPad Prism version 9.5 (GraphPad Software, San Diego, CA, USA).

## Figures and Tables

**Table 1:** Frequencies of inclusion (%) and dietary content (percentage of the diet on a DM basis) of the main ingredients incorporated in total mixed rations of investigated Mexican dairy farms.

Dietary Ingredient	Frequency of Inclusion (*n* = 19) (%)	Dietary Content (% of DM Basis)		
		Average ± SD	Median	Range
Whole-plant corn silage	100	38.9 ± 6.62	40.0	27.5–53.0
Alfalfa hay	79	9.1 ± 4.36	9.0	3.5–16.5
High-energy density concentrate	74	45.5 ± 8.85	48.5	28.3–60.0
Protein-rich concentrate	26	22.6 ± 3.58	25.0	17.0–25.0
Corn stover	21	3.5 ± 2.58	2.9	1.0–7.0
Rolled corn	21	25.5 ± 6.26	27.9	16.3–30.0
Corn meal	16	24.2 ± 9.80	23.0	15.0–34.5
Oat hay	16	3.3 ± 0.76	3.5	2.5–4.0
Sorghum silage	11	13.0 ± 2.83	13.0	11.0–15.0
Corn bran	11	13.8 ± 0.35	13.8	13.5–14.0
Alfalfa silage	5	–	–	5.0
Bakery byproduct	5	–	–	7.0
Brewery spent grain	5	–	–	9.5
Forage	100	49.9 ± 4.42	50.0	40.0–60.0
Concentrate	100	50.1 ± 4.42	50.0	40.0–60.0

SD = Standard deviation; DM = Dry matter.

**Table 2:** Concentrations of metabolites detected in whole-plant corn silages and total mixed rations of Mexican dairy farms.

Concentration (µg/kg DM) ^1^		Group of Metabolites										
		Ergot Alkaloids	*Alternaria*	*Aspergillus*	*Fusarium*	*Penicillium*	Other Fungal Species	Mycotoxins	Total Fungal Metabolites	Unspecific Metabolites	Phytoestrogens	Plant Metabolites
Whole-plant corn silages (*n* = 19)	Average	2.24	56.4	154	8700	243	164	8710	9300	20,320	1740	2450
	±SD	2.09	60.4	292	6410	163	247	6340	6620	4950	3290	3820
	Median	1.5	39.1	53.8	6460	193	84.8	6330	7670	19,920	638	726
	Minimum	0.1	9.10	2.5	39.6	28.7	12	79.5	219	9860	90.5	90.5
	Maximum	5.6	242	1040	22,110	520	1090	21,960	22,800	27,790	12,350	13,190
Total mixed rations (*n* = 19)	Average	3.98	48.7	61.7	5550	171	169	5590	6000	10,240	224,260	225,960
	±SD	5.88	33.8	46.5	3160	89.1	167	3000	3260	3200	129,040	129,040
	Median	1.4	44.5	58.4	5840	161.1	125.4	5970	6190	10,300	209,740	211,540
	Minimum	0.4	5.6	11.7	112	23.2	6.1	139	161	3540	34,270	35,910
	Maximum	12.7	108	179	10,510	337	697	10,570	11,070	15,980	448,670	449,400
Wilcoxon matched-pairs test	*p*-value *	0.625	0.418	0.7086	0.0005	0.0108	0.984	0.0006	0.0004	<0.0001	<0.0001	<0.0001

^1^ Values based on the sum of the concentrations of the metabolites of each respective group (see Table 3). * Significant differences between each set of matched pairs presented *p*-value < 0.05. SD = Standard deviation; DM = Dry matter.

## Data Availability

Data are available on request due to restrictions (data protection agreement).

## Supplementary Materials

The following supporting information can be downloaded at: https://www.mdpi.com/article/10.3390/toxins15020153/s1, Figure S1: Distribution of concentration (µg/kg DM, linear scale) of (**a**) groups of fungal metabolites and mycotoxins and (**b**) total phytoestrogens and plant metabolites detected in in whole-plant corn silages (yellow) and total mix rations (gray) in dairy farms in Mexico. Asterisks (*) show significant differences (*p*-value < 0.05) between the concentrations of the respective groups in whole-plant corn silages and total mixed rations according to the Wilcoxon matched-pairs signed rank test (*p*-values in Table 2). Means are shown as “+”; Table S1: Description of the co-contamination level of the diverse groups of analysis detected in whole-plant corn silages and total mixed rations of Mexican dairy farms; Table S2: *p*-values of the Spearman’s correlation coefficients (*ρ*) among groups of metabolites detected in total mixed rations with the main dietary ingredients. Significantly different (*p*-value < 0.05) presented in black cells; Table S3: List of 863 targeted metabolites to analyze whole-plant corn silages and total mixed rations from Mexican dairy farms via a validated multi-metabolite liquid chromatography/electrospray ionization–tandem mass spectrometry (LC/ESI–MS/MS); Table S4: Performance values of liquid chromatography/electrospray ionization–tandem mass spectrometry (LC/ESI–MS/MS) analysis for mycotoxins, phytoestrogens, and other fungal, plant, and unspecific metabolites detected in whole-plant corn silage and total mixed rations of dairy cattle in Mexico.
