# Correction: Penagos-Tabares et al. Mixtures of Mycotoxins, Phytoestrogens, and Other Secondary Metabolites in Whole-Plant Corn Silages and Total Mixed Rations of Dairy Farms in Central and Northern Mexico. *Toxins* 2023, *15*, 153

**DOI:** 10.3390/toxins16020062

**Published:** 2024-01-23

**Authors:** Felipe Penagos-Tabares, Michael Sulyok, Juan-Ignacio Artavia, Samanta-Irais Flores-Quiroz, César Garzón-Pérez, Ezequías Castillo-Lopez, Luis Zavala, Juan-David Orozco, Johannes Faas, Rudolf Krska, Qendrim Zebeli

**Affiliations:** 1 Unit of Nutritional Physiology, Institute of Physiology, Pathophysiology, and Biophysics, Department of Biomedical Sciences, University of Veterinary Medicine Vienna, Veterinärplatz 1, 1210 Vienna, Austria; 2 Christian-Doppler-Laboratory for Innovative Gut Health Concepts in Livestock (CDL-LiveGUT), Department for Farm Animals and Veterinary Public Health, University of Veterinary Medicine, Veterinaerplatz 1, 1210 Vienna, Austria; ezequias.castillo-lopez@vetmeduni.ac.at (E.C.-L.); qendrim.zebeli@vetmeduni.ac.at (Q.Z.); 3 FFoQSI GmbH—Austrian Competence Centre for Feed and Food Quality, Safety and Innovation, Technopark 1C, 3430 Tulln, Austria; 4 Department of Agrobiotechnology, IFA-Tulln, Institute of Bioanalytics and Agro-Metabolomics, University of Natural Resources and Life Sciences, Vienna, Konrad-Lorenz-Strasse 20, 3430 Tulln, Austria; michael.sulyok@boku.ac.at (M.S.); rudolf.krska@boku.ac.at (R.K.); 5 DSM-BIOMIN Research Center, Technopark 1, 3430 Tulln, Austria; ignacio.artavia@dsm.com (J.-I.A.); luis.zavala@dsm.com (L.Z.); juan-david.orozco@dsm.com (J.-D.O.); johannes.faas@dsm.com (J.F.); 6 Facultad de Estudios Superiores Cuautitlán, Cuautitlán, Medicina Veterinaria y Zootecnia, Universidad Nacional Autónoma de México (UNAM), Cuautitlán Izcalli 54714, Mexico; samantai.quiroz@gmail.com (S.-I.F.-Q.); smpda@comunidad.unam.mx (C.G.-P.); 7 Institute of Animal Nutrition and Functional Plant Compounds, Department of Farm Animals and Veterinary Public Health, University of Veterinary Medicine Vienna, Veterinaerplatz 1, 1210 Vienna, Austria; 8 Institute for Global Food Security, School of Biological Sciences, Queen’s University Belfast, 19 Chlorine Gardens, Belfast BT9 5DL, UK

## Error in Table

In the original publication [1], there was a mistake in Table 3 as published. A section of Table 3 (*Fusarium*-Metabolites) was unintentionally deleted during the edition process. The corrected Table 3 appears below.

The authors state that the scientific conclusions are unaffected. The original publication has also been updated.

## Figures and Tables

**Table 3:** Occurrences and concentrations of mycotoxins and other fungal metabolites detected in whole-plant corn silages and total mixed rations of Mexican dairy farms.

Group of Metabolites	Metabolite	Positive Samples ^1^ (%)	Whole-Plant Corn Silages (*n* = 19)			Positive Samples ^1^ (%)	Total Mixed Rations (*n* = 19)			Wilcoxon Matched-Pairs Test
			Concentration (µg/kg DM) ^2^				Concentration (µg/kg DM) ^2^			*p*-Value *
			Average ± SD	Median	Range		Average ± SD	Median	Range	
Ergot alkaloids	Festuclavine ^+^	5	–	–	2.41	0	–	–	–	>0.9999
	Dihydroergosine ^+^	26	1.35 ± 1.17	1.29	0.13–3.2	21	0.83 ± 0.97	0.44	0.18–2.28	0.0625
	Chanoclavine ^+^	5	–	–	2.04	5	–	–	12.5	>0.9999
*Alternaria* spp.	Altenuisol ^+^	32	2.5 ± 0	2.5	2.5–2.5	37	3.14 ± 1.7	2.5	2.5–6.99	0.7656
	Alternariol ^+^	5	–	–	5.5	11	9.77 ± 6.04	9.77	5.5–14	0.75
	Alternariolmethylether ^+^	47	9.89 ± 7.59	5.5	5.5–27.4	42	6.39 ± 2.51	5.5	5.5–12.6	0.25
	Altersetin ^+^	26	6.76 ± 4.5	5.16	1.25–12.7	42	15.7 ± 9.86	12.3	4.18–34.3	0.0488
	Infectopyron	21	97 ± 64	94	23.9–176	16	34.2 ± 3.38	36.1	30.3–36.2	0.1875
	Macrosporin ^+^	16	3.75 ± 0	3.75	3.75–3.75	11	3.75 ± 0	3.75	3.75–3.75	>0.9999
	Tentoxin ^+^	42	7.71 ± 4.86	6.38	3.1–16	79	6.91 ± 2.87	6.41	2.48–11.3	0.0932
	Tenuazonic acid ^+^	32	40.2 ± 10.4	37.5	30.1–60.4	53	49.4 ± 16.8	41.8	30.3–82.8	0.064
*Aspergillus* spp.	Averufin ^+^	42	3.6 ± 1.9	3.0	3.0–8.4	26	2.95 ± 0	2.95	2.95–2.95	0.125
	Deoxygerfelin	0	–	–	–	11	2.41 ± 1.33	2.41	1.47–3.35	0.5
	Flavoglaucin ^+^	11	2.8 ± 0.97	2.8	2.11–3.49	100	40.7 ± 29.6	41.6	3.63–111	<0.0001
	Fumigaclavine C ^+^	5	–	–	47.2	0	–	–	–	>0.9999
	Fumiquinazolin D ^+^	0	–	–	–	11	11.8 ± 5.34	11.8	8.01–15.6	0.5
	Kojic acid ^+^	11	877 ± 130	877	785–69	5	–	–	145	0.5
	Kotanin A	11	2.5 ± 0	2.5	2.5–2.5	5	–	–	2.50	>0.9999
	Methylsulochrin	5	–	–	4.5	11	4.5 ± 0	4.5	4.5–4.5	>0.9999
	Phenopyrrozin	84	56.1 ± 28.1	53.1	16.2–132	79	12.4 ± 5.14	10.7	7.16–24.1	<0.0001
	seco-Sterigmatocystin ^+^	16	2.72 ± 1.8	3.58	0.65–3.91	42	0.9 ± 0.46	0.65	0.65–1.71	>0.9999
	Sterigmatocystin ^+^	0	–		–	11	2.65 ± 0	2.65	2.65–2.65	0.5
	Versicolorin C	16	6.05 ± 3.98	3.75	3.75–10.6	0	–	–	–	0.25
*Fusarium* spp.	15-Acetyldeoxynivalenol ^+^	11	142 ± 46.7	142	109–175	0	–	–	–	0.5
	15-Hydroxyculmorin ^+^	32	2090 ± 1510	1580	464–4410	26	1270 ± 195	1280	993–1510	0.1563
	Acuminatum B ^+^	32	151 ± 89.6	142	58.3–290	26	52.2 ± 21.8	55.8	27.6–83.2	0.1094
	Antibiotic Y	5	–	–	9.5	5	–	–	9.5	>0.9999
	Apicidin ^+^	16	7.14 ± 2.32	7.23	4.78–9.41	5	–	–	9.04	0.5
	Aurofusarin ^+^	68	168 ± 386	48.8	3–1420	84	83.4 ± 67.3	67.1	11.4–224	0.2247
	Beauvericin ^+^	100	57.8 ± 74.7	32.3	5.46–330	100	33.1 ± 24.3	29.1	3.84–84.2	0.2266
	Beauvericin A ^+^	89	0.96 ± 1.55	0.45	0.45–6.87	84	0.55 ± 0.27	0.45	0.45–1.42	0.0204
	Bikaverin ^+^	95	224 ± 253	99.4	15.3–879	100	115 ± 95.6	94.7	18.1–308	0.0204
	Chrysogin ^+^	0	–	–	–	5	–	–	8.03	>0.9999
	Culmorin ^+^	58	865 ± 695	634	150–2090	58	505 ± 427	402	150–1420	0.0234
	Deoxyfusapyron	11	22 ± 12	22	13.5–30.5	16	591 ± 603	521	26.2–1230	0.375
	Deoxynivalenol ^+^	53	1500 ± 1080	1370	323–3350	84	615 ± 491	376	78–1670	0.1928
	DON-3-glucoside ^+^	26	74 ± 95.5	19.5	19.5–240	37	60.3 ± 23.5	65	19.5–86.6	0.3984
	Enniatin A ^+^	11	1.02 ± 1.15	1.02	0.2–1.83	37	0.45 ± 0.37	0.2	0.2–1.19	0.3438
	Enniatin A1 ^+^	11	0.4 ± 0	0.4	0.4–0.4	79	1.03 ± 0.82	0.4	0.4–2.6	0.0002
	Enniatin B ^+^	0	–	–	–	68	4.63 ± 5.62	1.4	1.4–18.8	0.0002
	Enniatin B1 ^+^	11	1.45 ± 0	1.45	1.45–1.45	89	3.99 ± 3.78	1.45	1.45–12.2	<0.0001
	Enniatin B2 ^+^	0	–	–	–	16	0.29 ± 0.05	0.29	0.24–0.34	0.25
	Epiequisetin ^+^	16	3.37 ± 1.6	3.78	1.6–4.72	5	–	–	1.6	0.375
	Equisetin ^+^	32	6.3 ± 6.27	3.05	1.6–14.7	42	5.19 ± 2.46	4.17	2.36–9.58	0.6836
	Fumonisin A1 precursor ^+^	16	63.2 ± 40.5	61.2	23.7–105	58	14.3 ± 14.1	9.16	3.55–48.9	0.3096
	Fumonisin A2 ^+^	11	43 ± 3.91	43	40.2–45.8	5	–	–	18	0.5
	Fumonisin B1 ^+^	47	723 ± 1050	124	26.5–2700	84	218 ± 244	126	26.5–1010	0.2288
	Fumonisin B2 ^+^	42	301 ± 371	72.6	18–987	68	103 ± 100	61.5	18–395	0.7722
	Fumonisin B3 ^+^	16	276 ± 145	297	121–409	32	57.1 ± 41.8	40	26.5–131	>0.9999
	Fumonisin B4 ^+^	16	78.3 ± 70.5	61	18–156	32	26.1 ± 20	18	18–66.9	0.5625
*Fusarium* spp.	Fungerin	0	–	–	–	5	–	–	26.5	>0.9999
	Fusaproliferin ^+^	37	403 ± 628	166	61.5–1820	58	280 ± 252	226	60.8–989	0.3054
	Fusapyron ^+^	5	–	–	1.5	5	–	–	5.46–5.46	>0.9999
	Fusaric acid ^+^	89	1210 ± 840	1130	260–3220	74	562 ± 235	503	298–1190	<0.0001
	Hydrolysed Fumonisin B1 ^+^	16	37 ± 49.1	10	7.29–93.7	5	–	–	30.4	0.75
	Moniliformin ^+^	89	88.9 ± 76.9	48	9–263	100	101 ± 67	78.8	27.6–247	0.1956
	Nivalenol ^+^	42	269 ± 184	209	103–614	68	872 ± 853	385	88.5–2600	0.0061
	Sambutoxin ^+^	37	0.37 ± 0.19	0.3	0.3–0.79	5	–	–	0.3	0.0625
	Siccanol ^+^	89	4620 ± 3530	3960	525–12,350	95	2510 ± 1650	2370	409–6130	0.0028
	W493	79	171 ± 190	80.7	3.55–694	74	86.6 ± 65.4	101	3.55–190	0.0256
	Zearalenone ^+^	68	58.7 ± 79.4	21.5	4.6–278	100	38.7 ± 57.2	17.8	4.6–246	0.9297
	Total enniatins	47	5.96 ± 7.24	1.60	0.60–19	89	11.2 ± 9.8	7.11	1.85–37	0.0144
	Total fumonisins	47	1150 ± 1570	203	26.5–4410	89	325 ± 396	155	3.6–1670	0.3867
	Total Type B trichothecenes	53	2000 ± 1230	1790	323–4230	89	1940 ± 1760	1156	78.0–5510	0.0505
*Penicillium* spp.	7-Hydroxypestalotin	53	17.3 ± 9.99	14.9	7.3–41.9	47	9.74 ± 4.87	9.74	2.6–16.7	0.0186
	Asterric acid	5	–	–	12.5	5	–	–	12.5	N/A
	Bilaid A	100	20.3 ± 22.9	11.4	5.78–87.6	95	8.53 ± 7.04	6.77	3.49–27.3	<0.0001
	Citreoviridin ^+^	0	–	–	–	21	42.9 ± 12.2	41.3	31.1–58	0.125
	Citrinin ^+^	0	–	–	–	5	–	–	77.9	>0.9999
	Cycloaspeptide A	0	–	–	–	5	–	–	13.4	>0.9999
	Cyclopenin	5	–	–	2.85	0	–	–	–	>0.9999
	Mycophenolic acid ^+^	11	90.2 ± 118	90.2	7–173	42	32 ± 42.9	11.4	7–127	0.1094
	Mycophenolic acid IV ^+^	5	–	–	2.53	0	–	–	–	>0.9999
	NP 1243	5	–	–	34.1	0	–	–	–	>0.9999
	Oxaline	16	68.9 ± 61.2	81.2	2.55–123	16	20.1 ± 14.9	12.9	10–37.2	0.5
	Pestalotin	53	29.2 ± 13.5	28.7	8.61–59.2	58	12.4 ± 6.93	11.2	3.3–24.5	0.0282
	PF 1163A	5	–	–	3.32	5	–	–	0.75	>0.9999
	Questiomycin	5	–	–	1.5	89	8.71 ± 7.72	8.6	0.6–23	<0.0001
	Questiomycin Derivate	95	184 ± 111	164	34.9–407	95	118 ± 64.2	106	18.1–238	0.0002
	Quinolactacin A	11	1.2 ± 0	1.2	1.2–1.2	21	1.2 ± 0	1.2	1.2–1.2	0.5
Other fungi	Ascochlorin	21	11.9 ± 10.2	8.43	3.75–26.9	21	6.24 ± 4.99	3.75	3.75–13.7	0.625
	Ascofuranone	21	2.26 ± 1.82	1.35	1.35–4.98	5	–	–	1.35	0.3125
	Bassianolide	37	3.17 ± 1.25	2.7	2.7–6	32	2.7 ± 0	2.7	2.7–2.7	0.5
	Beauveriolide I_III	26	1.5 ± 0	1.5	1.5–1.5	16	4.22 ± 3.01	3.71	1.5–7.45	0.6563
	Cercosporin	58	40.8 ± 25.6	36.5	13.2–87.9	79	72.2 ± 79.7	42.5	15.1–325	0.0479
	Cytochalasin J	0	–	–	–	11	136 ± 26.5	136	117–155	0.5
	Destruxin B ^+^	0	–	–	–	21	1.25 ± 0.68	1.1	0.7–2.09	0.125
	Ilicicolin A	5	–	–	6.23	37	1.83 ± 0.61	1.6	1.6–3.21	0.2813
	Ilicicolin B	79	18.9 ± 20.7	4.45	4.45–69.4	89	14.1 ± 9.96	13	4.45–28.9	0.6848
	Ilicicolin E	5	–	–	1.7	11	1.7 ± 0	1.7	1.7–1.7	>0.9999
	Monocerin	89	115 ± 237	37.4	2.1–990	74	85.9 ± 133	37.2	2.1–502	0.0024
	Mycousnine	0	–	–	–	11	0.75 ± 0	0.75	0.75–0.75	0.5
	Myriocin ^+^	16	67.9 ± 52.1	48.1	28.6–127	32	44.6 ± 26.2	41.1	15.7–92.6	0.5625
	Phomalone	5	–	–	6.14	0	–	–	–	>0.9999
	Sporidesmolide II	84	7.9 ± 13.2	2.92	0.75–44.7	74	4.4 ± 5.07	2.54	0.75–17.2	0.0643
	Sporidesmolide III	5	0.75	0.75	0.75	0	–	–	–	>0.9999
Unspecific metabolites	3-Nitropropionic acid	21	63 ± 60.9	43	18.5–147	21	18.5 ± 0	18.5	18.5–18.5	0.5
	Asperglaucide	5	–	–	5.99	100	27.3 ± 33.6	10.8	2.05–142	<0.0001
	Asperphenamate	5	–	–	4.89	79	5.98 ± 7.37	3.35	1.93–31.4	<0.0001
	Brevianamid F	89	171 ± 77.8	166	61–408	89	116 ± 40.6	112	49.2–228	0.0021
	Chrysophanol	47	226 ± 111	231	62.5–367	32	176 ± 65.1	205	62.5–226	0.0195
	Citreorosein	53	24.1 ± 12.4	19.1	14.7–54.4	37	19.1 ± 6.67	15.8	12.5–30.1	0.123
	Cyclo(L-Pro-L-Tyr)	100	4680 ± 2300	4570	926–8970	100	2180 ± 1110	1890	589–5360	0.0006
	Cyclo(L-Pro-L-Val)	100	14,760 ± 3820	13,450	6890–2200	100	7080 ± 2300	6790	2160–11,570	<0.0001
	Emodin	95	9.62 ± 5.31	9.22	3.5–23.1	95	46.9 ± 102	8.49	3.5–422	0.2312
	Fellutanine A	95	128 ± 51.8	127	48.8–260	89	94.3 ± 38.7	86.7	34.8–199	0.0053
	Iso-Rhodoptilometrin	58	1.58 ± 0.59	1.4	1.4–3.35	53	1.4 ± 0	1.4	1.4–1.4	0.5
	N-Benzoyl-Phenylalanine	0	–	–	–	21	12.2 ± 2.47	12.1	9.56–15	0.125
	Neoechinulin A	0	–	–	–	100	133 ± 78.4	102	29.6–304	<0.0001
	Norlichexanthone	5	–	–	1.9	47	1.9 ± 0	1.9	1.9	0.0078
	Rugulusovine	100	355 ± 153	373	137–681	100	204 ± 93.8	197	53.5–407	<0.0001
	Skyrin	68	2.06 ± 1.07	1.85	0.55–3.96	89	4.42 ± 6.13	2.48	0.55–27	0.0097
	Ternatin	5	–	–	6.32	0	–	–	–	>0.9999
	Tryptophol	42	258 ± 126	170	170–456	32	963 ± 817	642	170–2100	0.5508
Phytoestrogens	Biochanin	5	–	–	147	79	36.3 ± 13	34.5	20.2–61.6	0.0081
	Coumestrol	26	56 ± 104	8	8–241	89	157 ± 126	109	45.5–479	0.0011
	Daidzein	37	263 ± 351	89	89–1020	100	12,700 ± 6710	10,710	3820–27,620	<0.0001
	Daidzin	68	428 ± 719	191	91–2730	100	63,690 ± 40,170	65,640	9350–125,770	<0.0001
	Genistein	58	153 ± 272	47	47–947	100	11,760 ± 6170	11,190	3990–26,530	<0.0001
	Genistin	63	1000 ± 1850	362	110–6700	100	118,150 ± 75,850	113,270	157,180–249,320	<0.0001
	Glycitein	5	–	–	324	89	4790 ± 1840	4450	2220–8220	<0.0001
	Glycitin	11	364 ± 292	364	158–570	100	13,340 ± 7920	12,070	1080–27,390	<0.0001
	Ononin	5	–	–	46	100	176 ± 28	153.3	46–512	<0.0001
Other plant metabolites	Abscisic acid	42	1610 ± 2860	574	273–8670	100	1660 ± 636	1620	411–3270	0.0012
	Anisodamine	16	514 ± 373	470	164–907	16	137.2 ± 101	141	34.5–236	0.375
	Atropine	16	318 ± 85	360	219–374	11	69.1 ± 22.4	69.1	53.3–84.9	0.25
	Hyoscine	16	427 ± 391	473	15–794	11	215.7 ± 93.1	216	150–282	0.375
Bacterial	Nonactin	16	1 ± 0	1	1–1	26	1.3 ± 1.2	0.8	0.6–3.3	0.3906

^1^ Samples with values > limit of detection (LOD). ^2^ Excluding data < LOD. In case values > LOD and < limit of quantification (LOQ), LOQ/2 was used for calculation. * Significant differences between each set of matched pairs presented *p*-value < 0.05. SD = Standard deviation; DM = Dry matter; ^+^ = metabolites classified as mycotoxins.
