# Supplementary material for: Mixtures of Mycotoxins, Phytoestrogens, and Other Secondary Metabolites in Whole-Plant Corn Silages and Total Mixed Rations of Dairy Farms in Central and Northern Mexico
Source: Toxins (Basel). 2023 Feb 13;15(2):153. doi: 10.3390/toxins15020153 (PMC9965745; doi:10.3390/toxins15020153)
Supplement: Supplementary file 1 [file toxins-15-00153-s001.zip › toxins-2220010-supplementary.pdf]

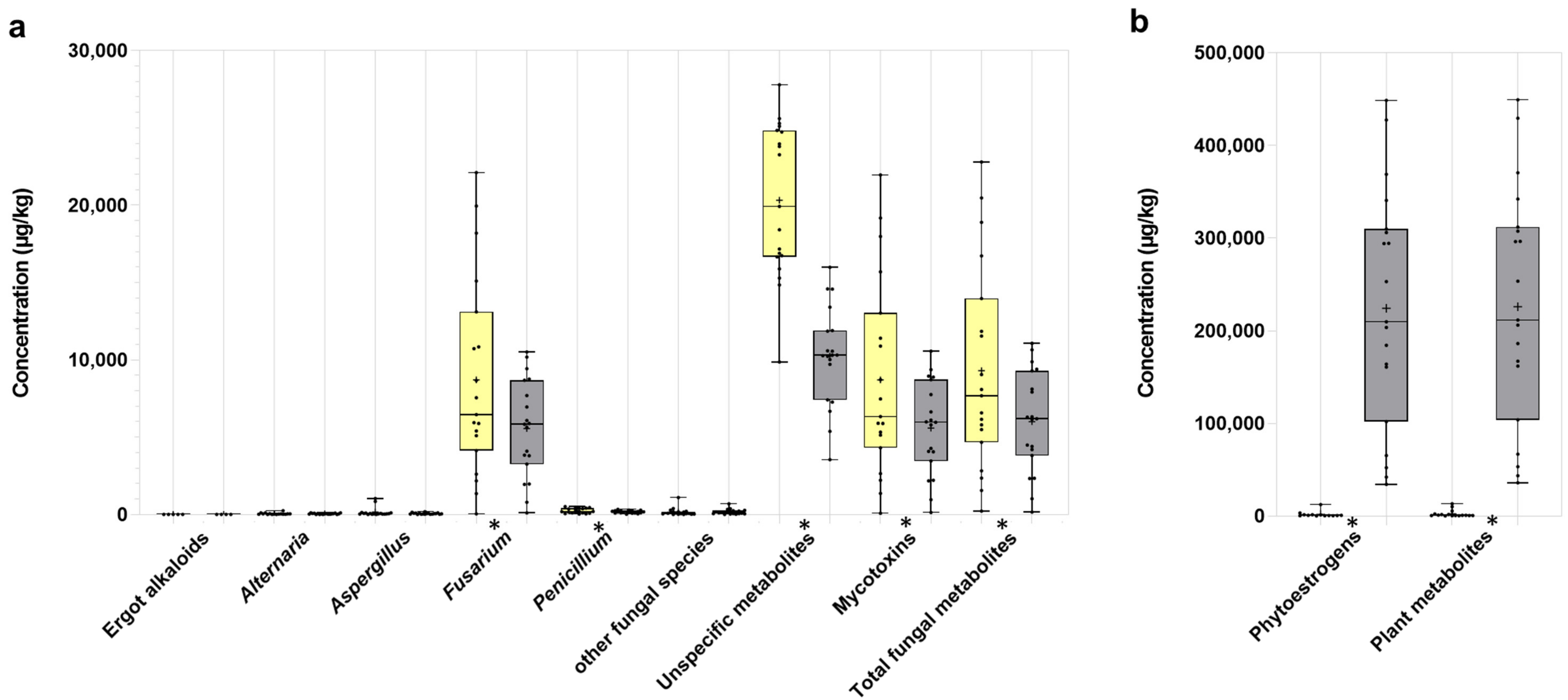

Figure S1. Distribution of concentration (µg/kg DM, linear scale) of (**a**) groups of fungal and unspecific metabolites, included accumulated mycotoxins and (**b**) total phytoestrogens and plant metabolites detected in whole-plant corn silages (yellow) and total mix rations (gray) in dairy farms in Mexico. Asterisks (*) show significant differences (p-value < 0.05) between the concentration in the re-spective group in whole-plant corn silages and total mix rations according to the Wilcoxon matched-pairs signed rank test (p-values in Table 2). Means are shown as "+".

Table S1. Description of the co-contamination level of the diverse groups of analysis detected in whole-plant corn silages and total mixed rations of Mexican dairy farms.

Group of metabolites	Whole-Plant Corn Silages (*n*=19)			Total Mixed Rations (*n*=19)			Wilcoxon Matched Pairs Test
	Co-Contamination (Metabolites/Sample)			Co-Contamination (Metabolites/Sample)			
	**Average ± SD**	**Median**	**Range**	**Average ± SD**	**Median**	**Range**	***p*-value***
Ergot alkaloids	1 ± 0.5	1	1 - 2	1 ± 0.5	1	1 - 2	0.5000
Alternaria	3 ± 1.7	2	1 - 6	3 ± 1.6	3	1 - 6	0.1624
Aspergillus	2 ± 1.3	2	1 - 5	3 ± 1.6	3	1 - 5	0.0034
Fusarium	14 ± 5.1	15	4 - 20	18 ± 4.9	20	5 - 24	0.0001
Penicillium	4 ± 1.2	4	2 - 6	5 ± 1.4	5	3 - 7	<0.0001
Other fungi	5 ± 1.3	4	3 - 7	5 ± 1.6	5	2 - 9	0.1865
Mycotoxins	17 ± 6.1	16	6 - 27	24 ± 5.6	26	9 - 31	<0.0001
Total fungal metabolites	27 ± 7.0	27	13 - 39	35 ± 7.8	36	13 - 43	<0.0001
Phytoestrogens	4 ± 1.8	4	1 - 8	9 ± 0.7	9	7 - 9	<0.0001
Plant metabolites	4 ± 2.4	3.5	1 - 9	9 ± 1.1	9	7 - 12	<0.0002
Unspecific metabolites	9 ± 1.7	9	6 - 12	12 ± 2.0	12	8 - 16	<0.0001
Total metabolites	29 ± 6.8	29	13 - 39	55 ± 8.9	58	31 - 66	<0.0001

* Significant differences between each set of matched pairs presented *p*-value < 0.05.

Table S2. P-values of the Spearman's correlation coefficients (ϱ) among groups of metabolites detected in total mixed rations with the main dietary ingredients. Significantly different (*p*-value < 0.05) presented in black cells.

Dietary Ingredients \ Groups of Metabolites	Ergot alkaloids	*Alternaria*	*Aspergillus*	*Fusarium*	*Penicillium*	Other fungal species	Unspecific metabolites	Type B trichothecenes	Fumonisins	Enniatins	Zearalenone	Mycotoxins	Fungal metabolites	Plant metabolites
**Forage**	0.0038	0.6430	0.6586	0.5071	0.1645	0.9201	0.6482	0.9700	0.2984	0.9614	0.4243	0.5071	0.4636	0.3339
**Concentrate**	0.0038	0.6430	0.6586	0.5071	0.1645	0.9201	0.6482	0.9700	0.2984	0.9614	0.4243	0.5071	0.4636	0.3339
**Whole-plant corn silage**	0.3456	0.1329	0.8752	0.6520	0.2807	0.4901	0.6807	0.6557	0.9275	0.8426	0.9684	0.6728	0.6755	0.8109
**Alfalfa hay**	0.6490	0.3118	0.3686	0.9713	0.4074	0.0859	0.7513	0.9613	0.1740	0.4986	0.9740	0.9857	0.9541	0.8998
**Alfalfa silage**	0.6227	0.7261	0.5984	0.4810	0.8611	0.8611	0.1489	0.1487	0.1234	0.4800	0.4771	0.4810	0.4810	0.7261
**Corn stover**	0.2784	0.6961	0.7794	0.0173	0.6374	0.8337	0.6961	0.3622	0.8180	0.8333	0.1450	0.0220	0.0305	0.3409
**Rolled corn**	0.8664	0.1551	0.6665	0.7640	1.0000	0.4620	0.5533	0.8493	0.6701	0.2526	0.3747	0.8415	0.9045	0.6886
**Corn meal**	0.5531	0.3076	0.6980	0.5729	0.3695	0.8574	0.7190	0.3273	0.8662	0.2833	0.9684	0.4345	0.4211	0.6122
**High-energy density concentrate**	0.5426	0.6755	0.3353	0.9684	0.8177	0.6492	0.9770	0.2471	0.6767	0.1527	0.7024	0.9426	0.9828	0.6207
**Sorghum silage**	0.4728	0.8830	0.0196	0.1433	0.2291	0.8619	0.9254	0.1826	0.0303	0.2336	0.0584	0.1828	0.2347	0.3084
**Protein-rich concentrate**	0.4614	0.4771	0.5281	0.7395	0.6570	0.1582	0.8500	0.1225	0.4581	0.5567	0.5182	0.6171	0.6370	0.7605
**Bakery by-product**	0.6227	0.1014	0.7261	0.2858	0.3764	0.8611	0.7261	0.8611	0.5982	0.2091	0.8599	0.2858	0.3764	0.2101
**Corn bran**	0.2351	0.1169	0.0856	0.7581	0.5452	0.5542	0.4338	0.2577	0.9893	0.8828	0.2717	0.8514	0.7479	0.0913
**Brewery's spent grains**	0.6227	0.2858	0.4810	0.7261	0.8611	0.2101	0.1014	0.2856	0.1487	0.8608	0.4771	0.7261	0.8611	0.7261
**Oat hay**	0.3639	0.4942	0.3217	0.7064	0.7148	0.0331	0.2378	0.8004	0.1143	0.0542	0.9503	0.6202	0.5537	0.0231

Table S3. List of 863 targeted metabolites to analyze whole-plant corn silages and total mixed rations from Mexican dairy farms via a validated multi-metabolite liquid chromatography/electrospray ionization-tandem mass spectrometry (LC/ESI–MS/MS).

10-Norparvulenone	Aflatoxin B1	Andrastin A	Aspterric acid
15-Acetyldeoxynivalenol	Aflatoxin B2	Andrastin B	Aspulvinone E
15-Desoxyoxalicine B	Aflatoxin G1	Andrastin C	Aspulvinone O
15-Hydroxyculmorin	Aflatoxin G2	Andrastin D	Aspyrone
15-Hydroxyculmoron	Aflatoxin M1	Andrastin Derivative	Asteltoxin
16-Ketoaspergillimide	Aflatoxin M2	Anisomycin	Asterric acid
1-Deoxypebrolide	Aflatoxin P1	Antbiotic L 696474	Asterriquinonedimethylether
2-Chlorunguinol	Aflatoxin Q1	Antibiotic F 1849 A	Aszonapyrone A
2-Methylmitorubin	Aflatrem	Antibiotic PF 1052	Atlantinon A
3,4,15Triacetylnivalenol	Aflavarin	Antibiotic Y	Atpenin A5
3,4-Diacetylnivalenol	Agistatin B	Apicidin	Atropine
3-Acetyldeoxynivalenol	Agistatin D	Apidicin C	Atroventinmethylether
3-Acetylneosolaniol	Agistatin E	Apidicin D2	Aurantiamin A
3-Acetyl-T-2 Toxin	Agroclavine	Aristolochic acid A	Auranticin A
3-Hydroxy-3-acetyl-T-2 Toxin	Aigualomycin D	Ascochlorin	Auranticin A
3-Hydroxy-HT-2 Toxin	AJ 296	Ascofuranone	Aurantine
3-Hydroxyterphenyllin	Alamethicin	Ascolactone	Aurantioclavin
3-Nitropropionic acid	alpha-Zearalenol	Ascomycin	Aurantiogliocladin
4,7,15Triacetylnivalenol	alpha-Zearalenol Glucoside	Asparason A	Aurasperon B
4-Hydroxyalternariol	Alteichin	Aspercolorin	Aurasperon C
4-Methoxycyclopeptin	Altenuene	Asperflavine	Aurasperon G
4-Monoacetoxyscirpenol	Altenuisol	Asperfuran	Aureobasidin
5-Hydroxyculmorin	Altenusin	Aspergamid A	Aurofusarin
5-Methylmellein	Alternarian acid	Aspergillicin Derivat	Austalide A
7-Hydroxykaurenolide	Alternarienoic acid	Aspergillimide	Austalide B
7-Hydroxypestalotin	Alternariol	Asperglaucide	Austalide Derivative
8-Acetylneosolaniol	Alternariol-3-Glucoside	Asperlactone	Austalide F
8-O-Methylaverufin	Alternariol-9-Glucoside	Asperloxine A	Austamide
A 23187	Alternariolmethylether	Aspermytin A	Austdiol
A 26771 B	Alternariolmethylether-Glucoside	Aspernigrin A	Austinol
AAL TA-Toxin	Altersetin	Asperphenamate	Austocystin A
AAL TB Toxin	Altersolanol	Asperthecin	Austocystin B
AAL TD Toxin	Altertoxin II	Aspinolid B	Austocystin D
AAL TE Toxin	Altertoxin-I	Aspinonene	Austocystin I
Abscisic acid	Amauromine	Aspochalasin C	Australide D
Acetylchaetoglobosin D	Amidepsin B	Aspochalasin D	Australide F
Achaetolide Derivat	Aminodimethyloctadecanol	Aspochalasin H	Averantin
Acuminatum B	Amoxycillin	Aspochalasin I	Averantinmethylether
Acuminatum C	Amphotericin	Aspochalasin J	Averufanin
Aflatoxicol	Anacin	Aspochracin	Averufin Derivat

Table S3. Cont.

Averufin	Cerulenin	Colchicine	Deepoxy-deoxynivalenol
Bacitracin	Chaconin	Communesin B	Deepoxy-T-2 toxin
Bafilomycin A1	Chaetocin	Cordycepin	Deepoxy-T-2tetraol
Banksialactone A	Chaetoglobosin A	Coumestrol	Dehydroaustinol
Barceloneic acid	Chaetoglobosin C	Culmorin	Dehydrocurvularin
Bassianolide	Chaetoglobosin D	Curvularin	Dehydrocyclopeptine
Beauvericin	Chaetoglobosin F	Curvulin	Dehydrogriseofulvin
Benzomalvin A	Chaetominine	Cyclo(l-Ala-L-Pro)	Demethoxyviridol
Benzomalvin B	Chaetoviridin A	Cyclo(L-Leu-L-Pro)	Demethylasteltoxin
Benzomalvin C	Chanoclavin	Cyclo(L-Pro-L-Tyr)	Demethylsulochrin
Berkedrimane B	Chetomin	Cyclo(L-Pro-L-Val)	DeoxyAltersolanol
Berkeleyacetal B	Chetoseminudin A	Cycloaspeptide A	Deoxybrevianamid E
Berkeleylctone E	Chevalone B	Cycloechinulin	Deoxyfusapyron
Berkeleylctone F	Chevalone C	Cycloheximide	Deoxygerfelin
beta-Zearalenol	Chevalone E	Cyclopenin	Deoxynivalenol
beta-Zearalenol-Glucoside	Chlamydospordiol	Cyclopenol	Deoxynortryptoquivalin
Bikaverin	Chlamydosporol	Cyclopeptine	Deoxytryptoquialanine
Biochanin	Chloramphenicol	Cyclopiazonsäure	Deoxytryptoquivaline A
Bis(methylthio)gliotoxin	Chlorocitreorosein	Cyclosporin A	Desoxypaxillin
Bongkrekic acid	Chloronectrin	Cyclosporin B	Desoxyverrucosidin
Botryodiplodin	Chlortetracyclin	Cyclosporin C	Destruxin A
Brasiliamide A	Chrodrimanin	Cyclosporin D	Destruxin B
Brefeldin A	Chromomycin A3	Cyclosporin H	Destruxin CHL
Brevianamid F	Chrysogin	Cylindrocarpon A4	Destruxin D
Brevicompanine B	Chrysophanol	Cylindrol B	Destruxin-Ed Derivat
Butenolid	Cinereanin	Cytochalasin A	Dethiosecoemestrin
Butyrolacton III	Citreohybriddione	Cytochalasin B	Diacetoxyscirpenol
Butyrolactone I	Citreohybridinol	Cytochalasin C	Diacetylcercosporin
Butyrolactone II	Citreoindole	Cytochalasin D	Diacetylnivalenol
ButyrolactonIImethylether	Citreorosein	Cytochalasin E	Dichlordiaportin
Byssochlamic acid	Citreoviridin	Cytochalasin J	Dichlormethylasterric acid
Calonectrin	Citreoviridin C	Daidzein	Diffractaic acid
Calphostin	Citreoviridinol	Daidzin	Dihydroaspyrone
Calphostin C	Citrinin	Daunorubicin	Dihydrochlamydocin
Calyxanthone	Citromycetin	Deacetylneosolaniol	Dihydrocitrinone
Carnequinazolin A	Cladosporin	Decalonectrin	Dihydrocompactin
Cephalochromin	Cladosporone Derivat	Dechlorogriseofulvin	Dihydroergosine
Cercosporamide	Clonostachydiol	Dechlorogriseofulvin	Dihydroergotamine
Cercosporin	CNM 115443	Dechloroisochromophilon IV	Dihydrogriseofulvin
Cereulide	Cochlioquinone A	Dechloronornidulin	Dihydroinfectopyron

Table S3. Cont.

Dihydrolysergol	Ergocristinine	Fumiquinazolin D	Glisoprenin D
Dihydrosterigmatocystin	Ergocryptine	Fumiquinazolin Derivat	Glyantrypine
Dihydrotrichotetronine	Ergocryptinine	Fumiquinazolin F	Glycitein
Dihydroxycalonectrin	Ergometrine	Fumitremorgin A	Glycitin
Dihydroxymellein	Ergometrinine	Fumitremorgin B	Grayanotoxin I
DihydroxyZONMethylether	Ergosin	Fumitremorgin C	Griseofulvin acid
Dinactin	Ergosinin	Fumonisin A1	Griseofulvin
Diplodiatoxin	Ergotamine	Fumonisin A1 (precussor)	Griseophenone A
DON-3-glucoside	Ergotaminine	Fumonisin A2	Griseophenone B
Doxorubicin	Ergovalin	Fumonisin AK2	Griseophenone C
Doxycyclin	Erucifolin	Fumonisin B1	Harzianopyridine
Drimane 6	Erucifolin-N-Oxid	Fumonisin B2	Harzianum A
Drimane 8	Erythromycin	Fumonisin B3	HC Toxin
Duclauxin	Ethylorsellinic acid	Fumonisin B4	Heliotrin
Echimidin	Europin	Fumonisin B6	Heliotrin-N-Oxid
Elymoclavine	Europin-N-Oxid	Fungerin	Helvolic acid
Elymoclavine-Fructoside	Expansolid	Fusaproliferin	Helvolinic acid
Emericellamide A	F01 1358-A	Fusapyron	Heptaibin
Emericellamide C	Fallacinol	Fusarenon-X	Heptelidic acid
Emericellamide E	FB1 Methylester	Fusaric acid	Herquline A
Emestrin	Fellutanine A	Fusarielin A	Hexaacetyl-HFB1
Emindole SA	Fellutannine B	Fusarin C	Hirsutide
Emodin	Festuclavine	Fusarinolic acid	HT-2 Glucoside
Endocrocin	Filipin	Fusarisetin A	HT-2 toxin
Enniatin A	FK 506	Fuscofusarin	hydrolysed Fumonisin B1
Enniatin A1	FK 9775 A	Galbinic acid	hydrolysed Nidulin
Enniatin B	FK 9775 B	Geldanamycin	Hydroxyandrastin A
Enniatin B1	Flavipucin	Genistein	Hydroxyandrastin C
Enniatin B2	Flavoglaucin	Genistin	Hydroxycarnequinazolin A
Enniatin B3	Folipastin	Geodin	Hydroxycurvularin
Epiequisetin	Fonsecin	Geodin hydrate	Hydroxypaspaline
Epoxyagroclavin	Formonetin	Gibberellic acid	Hydroxysulchorin
Epoxycytochalsin C	FS-4	Gibberellin A12	Hydroxysydonic acid
Equisetin	Fulvic acid	Gibberellin A14	Hyoscin
Eremofortin A	Fumagillin	Gibberellin A4	Hypothemycin
Eremofortin B	Fumarprotocetaric acid	Gibberrellin A7	Ilicicolin A
Ergine	Fumifungin	Gibepyron D	Ilicicolin B
Ergocornine	Fumigaclavine C	Gigantenone	Ilicicolin C
Ergocorninin	Fumigaclavine	Gliocladic acid	Ilicicolin E
Ergocristine	Fumiquinazolin A	Gliotoxin	Ilicicolin F

Table S3. Cont.

Ilicicolin H	Linamarin	Monocrotalin-N-Oxid	Ochrephilone
Indicin_IM_LA	Lincomycin	Monomethylcurvulin	Okaramine B
Indicin_IM_LA-N-Oxid	LL-Z 1272e	MPA Derivative	Okaramine D
Infectopyron	LLZ 1640-2	Mycophenolic acid IV	Oligomycin A
Integracin A	LLZ 1640-4	Mycophenolic acid	Oligomycin B
Integracin B	Lolitrem B	Myriocin	O-Methylsterigmatocystin
Ionomycin	Lolitrem N	Mytoxin C	O-Methylviridicatin
Irgasan	Lotaustralin	N-0352A	Ononin
Isocereulide A	Luteoskyrin	N-0532B	Ophiobolin A
Isochromophilon III	Luteusin A	N-Acetyl-HFB1	Ophiobolin B
Isochromophilon IV	Lysergol	N-Benzoyl-Phenylalanine	Orsellinic acid
Isochromophilon IX	Macrosphelide A	Neoechinulin A	Oxalicine B
Isochromophilone VI	Macrosporin	Neosartorin	Oxaline
Isofusidienol	Malformin A	Neosolaniol	Oxidized Elymoclavine
Isokotanin B	Malformin A2	Neoxaline	Oxidized Luol
Isopenicillide Derivative	Malformin C	NG 012	Oxisterigmatocystin E
Iso-Rhodoptilometrin	Marcfortine A	Nidulin	Oxyskyrin
Isosulochrin	Marcfortine C	Nidurufin	Papyracillic acid A
Jacobin	Meleagrin	Nigericin	Paracelsin A
Jacobin-N-Oxid	Meleagrin Derivative	Nigragillin	Paracelsin B
Janthitrem A	MER-NF5003E	Nigrosporoate A	Paraherquamide A
Josamycin	MER-NF5003F	Nivalenol Glucoside	Paraherquamide E
K252a	Methoxycurvularin	Nivalenol	Paspalic acid
K252b	Methoxysterigmatocystin	Nocardamine	Paspalin
K-76 Derivative 4	Methylasterric acid	Nonactin	Paspalinin
Kipukasin A	Methylequisetin	Norcitreoviridin	Paspalitrem A
Kipukasin B	Methylfunicone	Norlichexanthone	Paspalitrem B
Kipukasin D	Methylorsellinic acid	Nornidulin	Patulin
KO 143	Methylsulochrin	Norsolorinic acid	Paxillin
Kojic acid	Mevastatin	Norstictic acid	Pencillazaphilone B
Koninginin A	Mevinolin	Nortryptoquialanine	Penicillic acid
Koninginin B	Mithramycin C	Norverrucosidin	Penicillide
Koninginin D	Mitomycin	Notoamide Derivative	Penicillin G
Koninginin E	Mitorubinic acid	Notoamide E Derivat	Penicillin V
Kotanin A	Mollicellin D	NT-2 Toxin	Penicinoline
Kumbicin C	Monactin	Obscurolide A1	Penicolinate
Lasiocarpin	Moniliformin	Ochratoxin A	Penigequinolone A
Lasiocarpin-N-Oxid	Monoacetoxyscirpenol	Ochratoxin alpha	Peniprequinolone
Lecanoric acid	Monocerin	Ochratoxin B	Penitrem A
Leoidin	Monocrotalin	Ochratoxin C	Pennigritrem A

Table S3. Cont.

Pentahydroxyscirpenol	Quinadoline A	Sclerotiorin	Sydowinin A
Pentoxyfylline	Quinadoline B	Secalonic acid B	Sydowinin B
Pestalone	Quinolactacin A	Secalonic acid D	sydowinol
Pestalotin	Quinolactacin B	Secalonic acid F	T-2 Glucoside
Petromurin C	Quinolone A	Secoemestrin C Derivat	T-2 toxin
PF 1163 A	Radicicol	seco-Sterigmatocystin	T2-Tetraol
Phaseolinone	Radicinin	semi Xanthomegnin	T2-Triol
Phenopyrrozin	Radicinol	semi-Vioxanthin	Tanzawaic acid B
Phenylpyropene A	Radiclonic acid	Senecionin_Senecivernin	Taxol
Phomalone	Rapamycin	Senecionin_Senecivernin_N-Oxid	Tenellin
Phomopsidin	Rasfonin	Seneciphylline	Tensidol B
Phomopsin A	Retrorsin	Seneciphylline-N-Oxide	Tentoxin
Phomopsolide B	Roquefortine C	Senkirkin	Tenuazonic acid
Phomoxanthone A	Roquefortine D	Setusosin	Ternatin
Phthalexin	Roquefortine E	Siccanin	Terpendole C
p-Hydroxyphenopyrrozin	Roridin A	Siccanol	Terpendole E
Physcion	Roridin L2	Sissotrine	Terpendole I
Pinselin	Roritoxin C	Skyrin	Terphenyllin
Piscarinin A	Rorotoxin A	S-MethylDON	Terragine E
Porritoxinol	Rosellichalasin	Sorbicillacton A	Terrecyclic acid
Prehelminthosporol	Rubellin D	Sphingofungin B	Terrein
Prehelminthosporollacton	Rubratoxin A	Sphingofungin D	Terretonin
Prelaptin	Rubrofusarin	Spiculisporic acid	Terretonin F Derivat
Preussin	Rugulosin	Spiramycin	Territrem A
Pseurotin A	Rugulotrosin	Spirodihydrobenzofuranlactam IV	Territrem B
Puberulin A	Rugulovasine A	Sporidesmolide II	Tetraacetlnivalenol
Puromycin	Rugulusovine	Sporogen AO I	Tetraacetyl-T-2 Tetraol
Purpactin A	Salazinic acid	Stachybotryamide	Tetracycline
Purpuride	Sambucinol	Stachybotrylactam	Tetrahydrobostrycin
Pyranonigrin	Sartorypron	Stachybotrysin B	Thailandolide B
Pyrenocin A	Sartorypyrone B	Staurosporin	Thaxtomin A
Pyrenophorol	Satratoxin F	Stemphylperylenol	Thielavin B
Pyripyropene A	Satratoxin G	Sterigmatocystin	Toxoflavin
Pyripyropene B	Satratoxin H	Sticitc acid	Triacetoxyscirpenol
Pyripyropene D	Scalusamid A	Strobilactone A	Triacetyl-Deoxynivalenol
Pyrophen	Sch 725680	Sulochrin	Trichalasin B
Quadrone	Scirpentriol	Surfactin A	Trichodermamide C
Questiomycine A	Sclerotigenin	Surfactin B	Trichodermin
Questiomycine Derivat	Sclerotin A	Sydonic acid	Trichodesmin
Questiomycine	Sclerotioramin	Sydonol	Trichodimerol

Table S3. Cont.

Trichostatin A	Ustiloxin B	Violaceol II	Zinndiol
Trichotetronine	Ustiloxin D	Viomellein	Zinniamide
Trichothecin	Ustusol A	Vioxanthin	Zinniol
Trichothecolone	Valinomycin	Viridicatin	
Trichoverrin A	Vancomycin	Viridicatol	
Trypacidin	Vermistatin	Viridicatum toxin	
Tryprostatin A	Verrucarin A	Viriditoxin	
Tryprostatin B	Verrucarin J	Viridol	
Tryptophol	Verrucarol	Vulpinic acid	
Tryptoquialanine	Verrucofortine	W493	
Tryptoquialanine Derivat	Verrucosidin	WIN 68577	
Tryptoquivaline A	Verruculogen	WIN-64821	
Tryptoquivaline F	Verruculotoxin	Wortmannin	
Tryptoquivaline G	Versicolorin A	Xanthomegnin	
Tylosin	Versicolorin C	Xanthotoxin	
Ulocladol	Versiconal Acetat (Hemiacetal)	Xantocillin X1	
Unguinol	Versiconol	Yaequinolone J2	
Unugisin E	Verticillin A	Zearalenone	
Usnic acid	Violaceic acid	Zearalenone-14-glucoside	
Ustiloxin A	Violaceol I	Zeralenone-16-Glucoside	

Table S4. Performance values of liquid chromatography/electrospray ionization tandem mass spectrometry (LC/ESI–MS/MS) analysis for mycotoxins, phytoestrogens, other fungal, plant and unspecific metabolites detected in whole-plant corn silage and total mixed rations of dairy cattle in Mexico.

Metabolite	Apparent Recovery (%)	Recovery of Extraction (%)	LOD (µg/kg)	LOQ (µg/kg)
15-Acetyldeoxynivalenol	60	92	15.0	50.0
15-Hydroxyculmorin	77	85	3.00	10.0
3-Nitropropionic acid	41	86	11.0	37.0
7-Hydroxypestalotin	78	93	1.60	5.20
Abscisic acid	57	87	2.20	7.20
Acuminatum B	86	82	0.70	2.50
Altenuisol	100	100	1.50	5.00
Alternariol	45	94	3.40	11.0
Alternariolmethylether	52	67	3.30	11.0
Altersetin	210	98	0.70	2.50
Anisodamine	25	97	21.0	69.0
Antibiotic Y	95	88	5.70	19.00
Apicidin	65	69	0.50	1.50
Ascochlorin	42	95	2.30	7.50
Ascofuranone	41	95	0.80	2.70
Asperglaucide	83	99	1.20	4.10
Asperphenamate	75	88	0.10	0.40
Asterric acid	136	98	7.20	25.0
Atropine	66	94	8.90	30.0
Aurofusarin	75	100	2.00	6.00
Averufin	50	100	1.80	5.90
Bassianolide	58	99	1.60	5.40
Beauvericin	100	95	0.30	0.90
Beauvericin A	100	95	0.30	0.90
Beauveriolide I_III	75	100	1.00	3.00
Bikaverin	52	63	0.50	1.70
Bilaid A	75	100	1.00	3.00
Biochanin	47	92	5.00	15.0
Brevianamid F	49	81	2.40	8.10
Cercosporin	45	90	2.90	9.60
Chanoclavin	52	94	0.60	1.90
Chrysogin	92	97	0.70	2.40
Chrysophanol	47	91	38.0	125
Citreorosein	33	97	2.00	6.60
Citreoviridin	72	100	2.30	7.50
Citrinin	82	62	6.00	21.0
Citrinin	82	62	6.00	21.0

Table S4. Cont.

Metabolite	Apparent Recovery (%)	Recovery of Extraction (%)	LOD (µg/kg)	LOQ (µg/kg)
Coumestrol	100	100	5.00	15.0
Culmorin	72	91	100	300
cyclo(L-Pro-L-Tyr)	61	64	15.0	52.0
cyclo(L-Pro-L-Val)	53	85	1.40	4.60
Cycloaspeptide A	49	100	1.20	4.00
Cyclopenin	58	100	1.70	5.70
Cytochalasin H	66	92	2.50	8.20
Cytochalasin J	50	95	2.80	9.30
Daidzein	42	96	53.0	178
Daidzin	43	93	54.0	181
Deoxyfusapyron	68	94	1.10	3.40
Deoxygerfelin	75	100	0.20	0.60
Deoxynivalenol	83	95	3.60	12.0
Destruxin B	51	96	0.40	1.40
Dihydroergosine	56	63	0.02	0.07
DON-3-glucoside	100	87	12.0	39.0
Emodin	71	94	2.10	7.00
Enniatin A	80	89	0.10	0.40
Enniatin A1	93	91	0.20	0.80
Enniatin B	71	76	0.90	2.80
Enniatin B1	51	49	0.60	2.90
Enniatin B2	103	82	0.05	0.20
Epiequisetin	138	94	1.00	3.20
Equisetin	138	94	1.00	3.20
Fellutanine A	83	87	1.30	4.20
Festuclavine	57	97	0.60	1.90
Flavoglaucin	47	92	0.40	1.30
Fumigaclavine C	69	97	3.30	10.0
Fumiquinazolin D	53	96	1.00	3.20
Fumonisin A1 Vorstufe	126	82	2.10	7.10
Fumonisin A2	106	87	11.0	36.0
Fumonisin B1	119	88	16.0	53.0
Fumonisin B2	106	87	11.0	36.0
Fumonisin B3	111	94	16.0	53.0
Fumonisin B4	106	87	11.0	36.0
Fungerin	72	90	0.30	1.40
Fusaproliferin	100	100	10.0	30.0
Fusapyron	88	97	0.90	3.00
Fusaric acid	100	100	10.0	30.0
Genistein	62	92	28.0	93.0

Table S4. Cont.

Metabolite	Apparent Recovery (%)	Recovery of Extraction (%)	LOD (µg/kg)	LOQ (µg/kg)
Genistin	34	97	66.0	220
Glycitein	59	103	32.0	105
Glycitin	57	95	25.0	82.0
Hydrolysed Fumonisin B1	118	89	2.30	7.20
Hyoscine	64	90	10.0	30.0
Ilicicolin A	46	71	1.00	3.20
Ilicicolin B	36	99	2.70	8.90
Ilicicolin E	51	95	1.00	3.40
Infectopyron	69	86	2.00	6.70
Iso-Rhodoptilometrin	58	95	0.60	2.80
Kojic acid	58	89	87.0	290
Kotanin A	55	99	1.50	5.00
Macrosporin	58	98	2.20	7.50
Methylsulochrin	52	95	2.70	9.00
Moniliformin	97	69	5.30	18.0
Monocerin	67	99	1.30	4.20
Mycophenolic acid	88	98	4.30	14.0
Mycophenolic acid IV	84	94	0.40	1.20
Mycousnine	75	100	0.50	1.50
Myriocin	55	66	0.30	0.90
N-Benzoyl-Phenylalanine	87	99	2.00	6.50
Neoechinulin A	38	114	4.30	14.0
Nivalenol	74	96	1.10	3.60
Nonactin	87	96	0.20	0.50
Norlichexanthone	42	96	1.10	3.80
NP 1243	178	99	3.40	11.0
Ononin	53	95	28.0	92.0
Oxaline	101	100	1.70	5.10
Pestalotin	79	99	2.00	6.60
PF 1163A	100	100	0.50	1.50
Phenopyrrozin	111	91	0.80	2.60
Phomalone	57	90	1.60	5.40
Questiomycin	100	100	1.00	3.00
Questiomycin Derivat	100	100	1.00	3.00
Quinolactacin A	63	84	0.70	2.40
Rugulusovine	56	94	1.00	3.20
Sambutoxin	100	100	0.20	0.60
seco-Sterigmatocystin	62	87	0.40	1.30
Siccanol	111	95	7.00	23.0
Skyrin	84	96	0.30	1.10

Table S4. Cont.

Metabolite	Apparent Recovery (%)	Recovery of Extraction (%)	LOD (µg/kg)	LOQ (µg/kg)
Sporidesmolide II	100	100	0.50	1.50
Sporidesmolide III	100	100	0.50	1.50
Sterigmatocystin	57	89	1.60	5.30
Tentoxin	75	94	0.70	2.30
Tenuazonic acid	150	100	10.0	30.0
Ternatin	75	92	0.50	1.70
Ternatin	75	92	0.50	1.70
Tryptophol	30	99	100	340
Versicolorin C	33	93	2.30	7.50
W493	195	98	2.10	7.10
Zearalenone	70	91	2.80	9.20
